# Domain-general cognitive motivation: Evidence from economic decision-making – Final Registered Report

**DOI:** 10.1186/s41235-022-00363-z

**Published:** 2022-03-18

**Authors:** Jennifer L. Crawford, Sarah A. Eisenstein, Jonathan E. Peelle, Todd S. Braver

**Affiliations:** 1 Department of Psychological and Brain Sciences, Washington University in St. Louis, 1 Brookings Dr, Box 1125, St. Louis, MO 63130 USA; 2 Department of Psychiatry, Washington University in St. Louis, 660 South Euclid Avenue, Box 8225, St. Louis, MO 63110 USA; 3 Department of Radiology, Washington University in St. Louis, 660 South Euclid Avenue, Box 8225, St. Louis, MO 63110 USA; 4 Department of Otolaryngology, Washington University in St. Louis, 660 South Euclid Avenue, Box 8115, Saint Louis, MO 63110 USA

**Keywords:** Cognitive motivation, Listening effort, Working memory, Speech comprehension

## Abstract

Stable individual differences in cognitive motivation (i.e., the tendency to engage in and enjoy effortful cognitive activities) have been documented with self-report measures, yet convergent support for a trait-level construct is still lacking. In the present study, we used an innovative decision-making paradigm (COG-ED) to quantify the costs of cognitive effort, a metric of cognitive motivation, across two distinct cognitive domains: working memory (an *N*-back task) and speech comprehension (understanding spoken sentences in background noise). We hypothesized that cognitive motivation operates similarly within individuals, regardless of domain. Specifically, in 104 adults aged 18–40 years, we tested whether individual differences in effort costs are stable across domains, even after controlling for other potential sources of shared individual variation. Conversely, we evaluated whether the costs of cognitive effort across domains may be better explained in terms of other relevant cognitive and personality-related constructs, such as working memory capacity or reward sensitivity. We confirmed a reliable association among effort costs in both domains, even when these other sources of individual variation, as well as task load, are statistically controlled. Taken together, these results add support for trait-level variation in cognitive motivation impacting effort-based decision making across multiple domains.

**Supplementary Information:**

The online version contains supplementary material available at 10.1186/s41235-022-00363-z.

## Significance statement

In this study, we highlight the need to further investigate the construct validity of cognitive motivation using decision-making paradigms popularized in the neuroeconomics literature. Despite the growing popularity of behavioral and neuroeconomic paradigms for the study of cognitive motivation (i.e., cognitive effort), there is little work that assessed whether these metrics reflect stable individual differences in an individual’s propensity to engage in cognitively demanding activities. The study used an innovative cognitive effort discounting paradigm to test for the strength of association of cognitive motivation across two different cognitive domains while controlling for the influences of component processes that are thought to contribute to cognitive effort costs (e.g., working memory capacity). Importantly, the results suggest that decision-making patterns in this paradigm might be useful for identifying individual differences in cognitive motivation that relate to important life outcomes, such as academic achievement and/or the ability to seek out and scrutinize information pertinent to making every-day life decisions. Further, this work can provide the baseline of research needed to rigorously study the altered cognitive effort costs and decision making observed in psychopathology, as well as in both healthy and pathological aging.

## Introduction

People frequently make decisions regarding whether to engage in cognitively effortful activities (such as taking on a challenging project at work), or instead, choosing a less effortful alternative (such as mindlessly browsing the internet). Motivation is likely to serve as a key factor impacting the decision to engage in cognitively effortful activities. Indeed, when faced with the choice of whether to engage in a cognitively effortful activity, both the costs (e.g., how taxing is the activity) and benefits (e.g., how much will it improve career prospects) need to be weighed in order to come to a decision. Although cognitive effort-based decision making likely varies according to the particulars of any given situation, stable individual differences in motivation may also play an important role in the decision-making process. Whereas some individuals might tend to strongly weigh the costs of cognitive effort, choosing to forgo effortful activities more generally, others may welcome the challenges presented to them and engage in a multitude of cognitively demanding tasks in daily life. In other words, people appear to differ in the degree to which they have the motivation to engage with cognitively demanding tasks, or ideas.

Support for this trait-like tendency to engage in cognitively effortful activities has been found in personality psychology research. In particular, the construct of Need For Cognition, assessed via self-report questionnaire (Need for Cognition Scale; NCS), is conceptualized as a stable individual difference in the tendency to engage in, and enjoy, effortful cognitive activities (Cacioppo & Petty, 1982; Cacioppo et al., 1996) and is often referred to with the short-hand terminology of “cognitive motivation.” Likewise, individual differences in cognitive motivation, assessed via the NCS, have been found to relate to important life outcomes, such as academic achievement and the ability to seek out and scrutinize information pertinent to daily decision making (Cacioppo et al., 1996).

Taking a closer look at this definition, it is important to note the critical distinction between an individual’s need for cognition (i.e., cognitive motivation) and their cognitive abilities. Cognitive ability is well established as a major dimension of individual variation and is assessed both through general intelligence tests (e.g., standard IQ measures), as well as more specialized dimensions, such as fluid intelligence (which indexes novel problem-solving and reasoning ability) and working memory capacity (which indexes the degree to which information can be actively maintained in short-term storage and used towards on-going cognitive computation). Nevertheless, cognitive motivation has been conceptualized as a trait that operates distinctly from cognitive ability (Cacioppo et al., 1996), suggesting that it is a meaningful and unique construct in the study of individual differences. Indeed, empirical work supports this claim, demonstrating that an individual’s cognitive motivation is related to, but distinct from, their fluid intelligence (Fleischhauer et al., 2009; Hill et al., 2013) and working memory capacity (Hill et al., 2016; Therriault et al., 2015). Taken together, these findings provide support for the claim that cognitive motivation is a domain-general construct that indexes the propensity of an individual to engage in cognitively effortful activities independent of their cognitive and intellectual abilities**.**

Nevertheless, our current understanding of individual differences in cognitive motivation is constrained by limitations in the way that this construct has typically been assessed. Specifically, individual differences in cognitive motivation are almost exclusively reported using self-report measures, like the NCS (Cacioppo & Petty, 1982). Self-report measures have a number of well-recognized limitations, such as memory-related biases in retrospective reporting, susceptibility to demand characteristics, and social desirability concerns (Barrett et al., 1998). As a consequence of these well-recognized limitations, recent work has shifted the focus of investigation from self-report measures to sensitive behavioral indices of cognitive motivation, using methods from the field of behavioral economics. More specifically, these new developments place cognitive motivation within a decision-making framework in which cognitive motivation is measured using revealed preferences, reflecting the trade-off between the expected benefits and costs associated with engaging in cognitively effortful activities (Botvinick & Braver, 2015; Shenhav et al., 2013; Westbrook & Braver, 2015). For example, decision-making paradigms, such as the demand selection task (DST; Kool et al., 2010), have enabled the precise quantification of cognitive motivation using revealed preferences between performing tasks with more, or less, frequent task-switching, rather than using explicitly stated preferences; this work has demonstrated that individuals tend to avoid engaging in cognitive effort (Kool & Botvinick, 2014; Kool et al., 2010)**.**

Similar considerations have prompted the development, within our own group, of a novel decision-making paradigm known as the COG-ED (for cognitive effort discounting task; Westbrook et al., 2013). The COG-ED derives from other well-known discounting paradigms used in behavioral and neuroeconomics that have been used to examine how other cost factors such as delay, risk, or physical effort impact decision making regarding reward outcomes (Green & Myerson, 2013). For example, the EEfRT (effort expenditure for rewards task; Treadway et al., 2009) is a widely used physical effort-based decision-making task that has been shown to be sensitive to individual differences (Treadway et al., 2012a, 2012b) and clinical impairments, such as schizophrenia and depression (Barch et al., 2014; Treadway et al., 2012a, 2012b). Like the COG-ED, these tasks use decision-making trials to estimate the “point of subjective indifference” (or equivalence), in which two options are equally preferred, which can be used to determine how much a particular cost factor “discounts” the value of a given outcome. For example, in delay discounting paradigms, if an individual is found to equally prefer receiving $10 now to $25 in a month, then the 1-month delay is estimated to discount the reward value by $15.

In the COG-ED, the focus is on cognitive effort discounting, as participants make a series of decisions between low-effort, low-reward, and high-effort, high-reward options to identify their point of subjective indifference (Westbrook et al., 2013). The COG-ED has shown to be sensitive to individual differences in cognitive motivation: individuals higher in cognitive motivation, as indexed by the NCS, tend to choose performing cognitively effortful tasks more often than those with lower levels of cognitive motivation (Westbrook et al., 2013). Most recently, the COG-ED has also been examined in the domain of speech comprehension, to test the degree to which subjective effort is increased when trying to understand speech in the midst of background noise (McLaughlin et al., 2021). Both young and older adults discounted effortful listening, and in older, but not young adults, this was tied to both hearing ability and working memory capacity. Moreover, age differences in effortful listening still remained even when accounting for these ability factors, consistent with a role for cognitive motivation. Thus, the COG-ED offers a promising tool to test whether cognitive motivation operates as a trait-like construct across task domains and individuals.

Importantly, it has still not been rigorously tested whether the extant findings from the COG-ED and related behavioral economic paradigms reflect stable individual differences in the specific construct of cognitive motivation, rather than individual differences in other constructs, such as cognitive ability or other personality-related motivational indices (e.g., reward sensitivity). Furthermore, to date our understanding of individual differences in cognitive motivation has been limited due to testing this construct in just one task domain at a time. Thus, in order to more carefully test whether cognitive motivation indeed operates at a domain-general level, individual preferences need to be tested across multiple domains in order to de-confound them from the processes that underlie the cognitive tasks themselves, such as working memory capacity. Indeed, recent work has attempted to remedy these gaps in our understanding by assessing cognitive motivation across two different versions of the DST, in addition to collecting individual difference measures of cognitive motivation (e.g., NCS) and ability (e.g., trail making test; Strobel et al., 2020). Interestingly, this study found that both the behavioral and self-reported measures of cognitive motivation showed evidence of trait variance when controlling for cognitive ability; however, the two measures did not correlate with each other (Strobel et al., 2020). On the surface, these results seem to suggest that the behavioral paradigms aimed at assessing cognitive motivation do not map onto measures indexing the same construct via self-report. However, since this experiment only tested one type of economic decision-making paradigm (DST), the results leave open the possibility that the null findings reflected the particular paradigm used, and that other decision-making paradigms, such as the COG-ED, may provide more robust indices of the latent cognitive motivational construct.

Following up from this recent work, in the current study we aimed to test whether individual differences in participants’ cognitive motivation show strong relationships across distinct cognitive domains. More specifically, by using the COG-ED to quantify cognitive effort costs (in addition to assessment with the more traditional NCS), we examined whether individuals who exhibit high cognitive motivation, within the domain of working memory, also exhibit high cognitive motivation in the domain of speech comprehension. Thus, we assessed cognitive motivation in two distinct domains, both of which rely on some of the same cognitive processes (Peelle, 2018), to test whether cognitive motivation is a stable, domain-general trait that can be observed across multiple cognitive contexts, using a sensitive behavioral paradigm. Indeed, we predicted that we would observe a strong association between the costs of cognitive effort (i.e., cognitive motivation) in working memory and speech comprehension domains, suggesting that there is a stable, trait-like, cognitive motivational construct that contributes to an individual’s cognitive effort costs (Hypothesis 1). Moreover, even when controlling both for task performance in these two domains, as well as other relevant processes (e.g., working memory capacity, personality traits indexing reward motivation), we predicted that there would still be an association between cognitive effort costs across working memory and speech comprehension domains, providing stronger evidence for a domain-general cognitive motivational construct (Hypothesis 2).

## Methods

### Ethics information

All experimental procedures were approved by the Washington University Human Research Protections Office prior to data collection. Participants provided informed consent and were compensated $10/hour for all study procedures, with the opportunity to gain up to an additional $8 bonus, based on the experimental tasks.

### Pilot data

A sample of healthy adults (*N* = 31, 18–23 years old) completed a pilot study to assess the feasibility of completing cognitive effort discounting procedures across both working memory and speech comprehension domains (see A1 for further details). As a brief overview, participants completed a task familiarization phase in which they performed either a *N*-back task, with working memory load varied across blocks (i.e., how many previous items need to be stored in working memory; *N* = 1–4, with higher *N* indicating increased cognitive demands), or a speech-in-noise task, with effortful speech comprehension varied across blocks (i.e., listening to spoken sentences presented with different levels of background noise; signal-to-noise ratios [SNRs] ranging from − 12 to 0 dB, with lower numbers corresponding to greater cognitive demands). Following the familiarization phase, participants completed a decision-making phase, by performing the COG-ED in each of the two domains (i.e., *N*-Back, speech-in-noise). In the COG-ED, with conditions adapted from prior work (Westbrook et al., 2013), participants were required to make a series of decisions between performing high-effort task levels (e.g., 2, 3, 4 back; − 4, − 8, − 12 SNR) for high monetary reward or low-effort task levels (e.g., 1-back; 0 SNR) for a lower monetary reward value. Critically, a within-subject design was employed, with each participant completing both the familiarization and discounting phases in both working memory and speech comprehension domains (counterbalanced across participants).

This design enabled us to quantify the subjective costs of cognitive effort for each participant in each domain, and to look at relationships between them.

We found that across both domains, participants discount task load (i.e., cognitive effort) similarly, whereby more difficult levels of the task (i.e., purple; 4-Back, − 12 SNR) are discounted more, or have a lower subjective value, relative to easier task levels (i.e., red; 2-Back, − 4 SNR), *B* = − 0.15 [− 0.12, − 0.18], SD = 0.02, with no differences observed across domains, *B* = 0.08 [− 0.02, 0.17], SD = 0.05 (Additional file 1: Fig. S1). Furthermore, examining the average subjective value of cognitive effort across working memory and speech domains revealed a strong within-subjects association, *r* = 0.521 [0.234, 0.744], BF_10_ = 39.21. In other words, participants who exhibited a low subjective value of cognitive effort (i.e., find engaging in cognitive effort to be costlier) in the working memory domain also tended to have a low subjective value of cognitive effort in the speech comprehension domain (Additional file 1: Fig. S2). The relationship between the costs of cognitive effort in working memory and speech comprehension domains remained, even after controlling for individual differences related to task difficulty and performance in each respective domain (working memory: hit rate, correct rejection rate, mean RT; speech comprehension: intelligibility), *r* = 0.400 [0.213, 0.558].

Self-reported ratings of mental demand, effort, and frustration provided further support of the costs of cognitive effort in each domain. There was a main effect of task load across ratings of mental demand *B* = 13.95 [11.09, 16.73], SD = 1.43, effort *B* = 11.81 [9.09, 14.49], SD = 1.38, and frustration *B* = 8.49 [5.68, 11.31], SD = 1.43. This suggests that as task load level increased, subjective ratings of mental demand, effort, and frustration increased. However, in contrast to the behavioral findings, there was also a main effect of domain for self-reported ratings of effort *B* = − 15.81 [− 23.23, − 8.23], SD = 3.79, and mental demand *B* = − 10.37 [− 17.50, − 3.19], SD = 3.65, which indicated that participants rated the speech-in-noise task to be less mentally demanding and effortful overall, relative to the working memory task. Frustration ratings did not differ across task domain, *B* = − 0.39 [− 7.82, 7.00], SD = 3.82.

Furthermore, we did not find conclusive evidence for a relationship between self-reported (e.g., NCS) and behavioral measures of cognitive motivation (e.g., cognitive effort discounting) in the pilot sample. Correlations between NCS and the working memory COG-ED (*r* = 0.115 [− 0.218, 0.451], BF_10_ = 0.33), speech comprehension COG-ED (*r* = 0.174 [− 0.145, 0.493], BF_10_ = 0.45), and the composite COG-ED score (*r* = 0.158 [− 0.197, 0.471], BF_10_ = 0.42) were anecdotal. It is important to note that in the pilot data, other potential covariates, such as working memory capacity or personality traits, were not assessed.

### Design

To examine the relationship between experimental measures of cognitive effort, we used the COG-ED (Westbrook et al., 2013) to estimate the subjective value (i.e., cost) of cognitive effort across two domains (i.e., working memory, speech comprehension) and test for within-subject associations between these two domains. Moreover, we obtained individual difference measures of the component processes that seemed plausibly likely to contribute to the computation of the cognitive effort costs (i.e., working memory capacity, reward sensitivity). The assessment of these other measures provided the means to statistically control for their influence (via partial correlation) when assessing the strength of the association of cognitive effort discounting across working memory and speech comprehension domains.

Orthogonal to our main hypotheses of interest, we also collected a self-reported measure of cognitive motivation (NCS), in order to test for the strength of the association between self-reported and behavioral indices of cognitive motivation. Although the NCS assessment and analyses were not the primary scope of this experiment, collecting these data provided an important baseline of research needed to rigorously explore the relationships between self-reported and behavioral measures of cognitive motivation in future work.

The experiment took place via remote online testing, across two separate sessions, scheduled approximately 24 h apart. All questionnaires and tasks were self-administered using the software platform Inquisit 6 (www.millsecond.com). In the first experimental session, participants were assessed with a range of individual difference measures that indexed working memory capacity (Listening-span; L-span; Cai et al., 2015); Operation-Span; O-Span; Symmetry-Span; Sym-Span; Unsworth et al., 2005). In addition, we collected self-report measures of reward motivation: Behavioral Inhibition and Behavioral Activation Scales (BIS/BAS; Carver & White, 1994), Generalized Reward and Punishment Expectancy Scale (GRAPES; Ball & Zuckerman, 1990), and Sensitivity to Punishment and Sensitivity to Reward Questionnaire (SPSRQ; Torrubia et al., 2001). Self-reported cognitive motivation (NCS; Cacioppo & Petty, 1982) was also collected for use with exploratory analyses. All tasks and questionnaires during this session were administered in the same order across participants.

#### Working memory familiarization phase

In the second experimental session, participants completed the familiarization and decision-making phases of the COG-ED within each cognitive domain. During the familiarization phase, participants first experienced variously demanding levels of either the working memory or speech-in-noise task; task order was fixed across participants. Both familiarization blocks (working memory, speech comprehension) were roughly equated in total duration. In the working memory task (*N*-Back), participants respond to each of a sequence of letters, presented one at a time in the center of a computer screen**.** The task requires that participants indicate when the current stimulus (i.e., letter) matches the letter from *N* steps earlier in the sequence (target) or when the stimulus differs from the letter presented *N* steps earlier (non-target). Prior work has shown that as the level of *N* increases, the task becomes progressively more difficult and effortful (Ewing & Fairclough, 2010). Participants completed one 20-trial run (5 targets; 15 non-targets) of each level of the task (1-back, 2-back, 3-back, 4-back) in ascending order of difficulty. Each level of the task was assigned a color (i.e., 2-Back = “red”) to avoid anchoring effects (i.e., cognitive biases that could cause subjects to base judgments off of an initial level of difficulty; Ariely et al., 2003). Thus, participants learned to associate each task level with its assigned color before beginning the discounting procedure. This discounting procedure has been successfully used across multiple participant populations, showing robust effects (Culbreth et al., 2019; Westbrook et al., 2013). *N*-back task performance during this familiarization phase was assessed in terms of hit rate, correct rejection rate, and mean RT for each task load level. As described below, these values were used to statistically control for individual differences in task performance when estimating cognitive motivation in the working memory domain.

#### Speech comprehension familiarization phase

During the speech-in-noise task, adapted from McLaughlin et al. (2021)**,** participants were presented with sentences with varying levels of noise. We used speech-shaped noise, that is, steady noise with a spectrum matching that of the sentences. Prior to starting the experiment, participants were encouraged to locate to a quiet space and use headphones for the task, if possible. The signal-to-noise ratio (SNR) was adjusted to manipulate task difficulty; negative SNR values indicate that the signal is presented at a lower level than the noise. Sentences were presented at various levels of noise (SNRs of 0 dB, − 4 dB, − 8 dB, and − 12 dB), and participants were instructed to type the sentence they heard back into a text box on each trial. If they were unsure of any words in a sentence, they were instructed to make their best guess. Each task level consisted of 15 self-paced trials wherein participants heard a sentence, typed it back into a text box, and then used the spacebar to begin the next trial. Like the working memory task, participants completed task blocks in order of difficulty, from easiest (0 dB SNR) to hardest (− 12 dB SNR), with the same color mappings for task difficulty used in the working memory task. Speech task performance during this familiarization phase was assessed in terms of intelligibility, operationalized as the number of key words in each sentence that were entered correctly (each sentence included four key words). As described below, these values were used to statistically control for individual differences in task performance when estimating cognitive motivation in the speech comprehension domain.

Following each run of the familiarization task (i.e., completing the 1-Back or 0 SNR task), participants completed self-reported ratings of the mental demand, physical demand, temporal demand, effort, frustration, and performance from the preceding task block using the NASA Task Load Index (Hart, 2006). Participants provided their responses using a visual analog scale ranging from 1 (very low)—21 (very high). These ratings helped to serve as a manipulation check to ensure that participants found the tasks to be effortful and mentally demanding across each load level.

#### Discounting phase

After the familiarization phase, in which each load level was experienced and practiced, the critical decision-making phase of the COG-ED occurred. In this phase, participants made repeated choices about whether to repeat performance of a higher load-level of the task (e.g., 2-, 3-, or 4-back; − 4, − 8, or − 12 SNR) or instead perform the easiest load level (1-back, 0 SNR). In the first trial of each higher- and low-effort pairing, participants were presented with equal reward amounts (either $2, $3, or $4) for completing the chosen task (e.g., $2 for 1-back vs. $2 for 2-back). The offer for the chosen task was then stepwise titrated across a series of 5 calibration trials, to estimate the value at which participants were indifferent between the two offers (i.e., they would be equally likely to choose either offer). For example, if a participant chose the $2 for 1-Back over $2 for the 2-Back, then the next calibration trial would present the participant with the offer of performing the 1-Back for $1 (i.e., half of the amount of the previous offer) or performing the 2-Back for $2 (i.e., fixed offer amount). On the other hand, if the participant instead chose to perform the 2-Back for $2 on the first trial (relative to $2 for the 1-Back) then the offer amount for the higher effort option would be stepwise titrated until the indifference point was reached. The point of subjective indifference is critical because it quantifies how much more subjectively costly the unchosen task level is relative to the chosen task. As a result, these indifference points estimate the “cost” of cognitive effort. In other words, the indifference point is the amount of money an individual is willing to forgo to avoid performing the unchosen task.

Participants completed a total of 45 decision trials in each domain (3 task load levels × 3 monetary reward levels × 5 calibration trials, with the task load and reward levels randomly intermixed) after they completed the corresponding familiarization phase. Critically, participants were informed that one of their choices would be used to determine task-based compensation and that they would be asked to repeat the task they chose, for the amount of money offered (i.e., $2 for the “red” task). Task-based compensation was not based on performance from the familiarization phase, but rather, participants were told that in order to successfully earn the money for repeating the chosen task, they would need to maintain their effort from the familiarization block when repeating the task block.

After completing all task blocks in each domain, participants completed a post-task questionnaire to assess how much their choices during the discounting phase were based on the difficulty, effort, or monetary reward associated with the task. In addition, after completing the speech comprehension phase, participants were asked what device was used to complete the task (e.g., speakers, headphones). Complete descriptions of all self-report questionnaires are provided in Additional file 1. Data collection and analysis were not performed blind to the conditions of the experiments.

### Power analysis

We used Bayes factor design analysis (BFDA) to determine the sample size for this experiment. Adopting a sequential design with maximal *N* using BFDA helped to ensure that we were collecting sufficient evidence while maintaining efficiency in our design (Schönbrodt & Wagenmakers, 2018; Schönbrodt et al., 2017). As an overview, in sequential designs, sampling is continued until the desired level of the strength of evidence is reached (i.e., Bayes factor; BF_10_), which in this case is 10 times in favor of the experimental hypothesis over the null hypothesis, or vice versa. To strike a balance between the feasibility and interpretability of the results, we planned to stop all data collection after the maximal *N* for this study (*N* = 300) was collected, if the Bayes factor threshold had not already been reached. To aid in the calculation of the approximate sample size, we used the *BFDA* package (Schönbrodt & Stefan, 2019), which runs 10,000 Monte Carlo simulations based on the pre-specified prior distribution and effect size estimates provided by the user. For this experiment, we opted to follow the approach of a safeguard power analysis (Perugini et al., 2014), choosing a smaller effect size (*r* = 0.3) than what was previously observed in our pilot study (*r* ~ 0.5 or *r* ~ 0.4 after controlling for task performance) in order to avoid underestimating the sample size. Furthermore, we decided to use an uninformed prior, a central Cauchy distribution with a scaling parameter of *r* = √2/2, as is default in the *BayesFactor* (Morey & Rouder, 2018) package in R, taking a more conservative approach to power analysis.

Results from the simulations suggested that the median sample size needed to obtain a Bayes factor ≥ 10 given the parameters specified above was *N* = 112, and, conversely, finding evidence in support for the null hypothesis, BF_10_ ≤ 0.1, would require a median sample size of *N* = 140 (results summarized in Additional file 1). Thus, we planned to sample, at minimum, 100 participants; after reaching this sample size, we then tested for sufficient evidence every ten participants thereafter, until the Bayes factor threshold (i.e., BF_10_ ≥ 10 or BF_10_ ≤ 0.1) was reached or until we collected data from 300 participants, the maximal N.

### Sampling

Participants were healthy adults, ages 18–40 years, recruited through the online research platform Prolific (www.prolific.co) (Palan & Schitter, 2018). Inclusion criteria for participation included English as native language, with no lifetime history of neurological trauma, seizures, hearing difficulty, or mental illness, and no current use of psychotropic medications. After completing the first experimental session indexing individual differences in working memory capacity and reward sensitivity, participants were invited back to participate in the second experimental session (e.g., discounting) if they completed all tasks and questionnaires from the first session (*n* = 184). From this sample, 52 participants declined to participate in the second experimental session. In addition, participants were excluded from the final sample if they reported not using headphones during the speech comprehension task (*n* = 10) or if they did not complete all parts of both discounting tasks (*n* = 18). The final sample consisted of 104 participants (47 females; 18–40 years, *M* = 27.3, SD = 5.8; 1 American Indian or Alaskan Native, 13 Asian, 11 Black or African American, 73 White, 5 more than 1 race, 1 not reported; 18 Hispanic or Latinx). We strived to use all available data in the subsequent analyses. However, a small subset of participants (*n* = 5) exhibited a behavioral profile that suggested possible non-compliance with the task instructions (e.g., almost always choosing the high-effort option; average subjective value > 1, or in other words, a reverse discounting pattern). As such, we performed additional analyses both with and without the excluded participant(s) and report both sets of values.

### Analysis

Bayesian linear mixed effect models were conducted in the package *brms* (version 2.16.1; Bürkner, 2017, 2018), R version 4.1.0 (RRID:SCR_001905; R Core Team, 2018) to estimate the effects of task load, domain (e.g., working memory, speech comprehension), and performance variables (*N*-Back: hit rate, correct rejection rate, mean RT; speech: intelligibility) on participants' discounting behaviors. Additional analyses estimated the effects of task load and domain on participants’ self-reported ratings of mental demand, effort, and frustration. In all models, task, domain, and performance variables were entered as fixed effects, with a random effect of intercept. Further, we used the default prior distributions in *brms* for each of the fixed effects (i.e., flat prior; central t-distribution, *df* = 3) and default number of iterations (4000) for each of these models, providing an estimate equivalent to maximum likelihood approaches used in multilevel modeling (using the package *lme4*; Bates et al., 2015). In the reported results, we provide the beta estimate (i.e., mean of the posterior distribution), the 95% credible intervals, standard deviation of the posterior distribution (i.e., error), and a Bayesian approximation of R^2^ (for more information, see Gelman et al., 2018).

The main variable of interest for our analysis was the subjective value (i.e., cost) of cognitive effort. The subjective value was calculated using each participant’s responses during the discounting procedure; as an overview, participants made repeated choices between high- and low-effort tasks, each at equal offer amounts at fixed values ($2, $3, $4), and the monetary values of the chosen option (either high- or low-effort task) were then stepwise titrated across a series of 5 calibration trials, with each trial in the series utilizing the participant’s prior responses to set the current value. The value of the titrated reward at the end of the calibration series, provided the indifference point (i.e., the value at which the participant was equally likely to choose either the low- or high-effort option) for a given amount and task load pairing. For task choices following trials in which participants initially chose the low-effort option (e.g., discounting high-effort option), each indifference point was divided by the corresponding monetary value of the high-effort option either $2, $3, or $4, to summarize the subjective value of engaging in cognitive effort, a positive value ranging from 0 to 1. If participants initially choose the high-effort option when presented with equal monetary rewards for performing the high- or low-effort task (i.e., discounting the low-effort option), we subtracted the indifference point from the fixed monetary reward amount and divided by the value of the fixed monetary reward. We transformed all subjective value estimates in which participants initially chose the high-effort option by adding 1 to the estimate, such that the subjective value estimate ranged from 0 to 2; values > 1 indicate preferences for higher effort tasks, whereas values < 1 indicate preference for the easy task.

The initial stage of analyses was to examine the subjective value estimates in each domain, in order to evaluate the effect of reward amount and task load factors. Additionally, we examined the effect of these factors on self-reported ratings of mental demand, effort, and frustration. In the first test of our hypothesis, we measured the zero-order correlation between cognitive effort discounting, estimated separately from the working memory and speech comprehension domains. For this analysis, we first calculated the average subjective value across all task conditions (3 monetary reward amounts × 3 task load levels) for each participant in each domain, then using the *Correlation* package in R (Makowski et al., 2020), which implements Bayesian correlations using the package *bayestestR* (Makowski et al., 2019), we correlated those two subjective value estimates with each other. An uninformed prior was used for this analysis, Cauchy distribution (*µ* = 0, *r* = √2/2). We report the correlation value as the median of the posterior distribution, in addition to the 95% credible intervals. Further, we report the Bayes factor, which contrasts the strength of the experimental model (i.e., correlation between effort costs across domains) relative to the null hypothesis (i.e., no correlation between effort costs across domains). This analysis served to replicate the initial finding in our pilot sample, which showed a strong association between the subjective value of cognitive effort across working memory and speech comprehension domains.

For the second test of our hypothesis, we first statistically controlled for task load and performance in each respective domain prior to computing the correlation between cognitive effort discounting in working memory and speech comprehension domains. To accomplish this, we entered task-level and relevant task performance variables (*N*-Back: hit rate, correct rejection rate, mean RT; speech: intelligibility) as covariates in a multilevel model predicting subjective value in each domain separately using the package *brms* in R (Bürkner, 2017, 2018); the averaged residuals from each participant within each domain were then correlated with each other using the same uninformed prior distribution as detailed above in order to quantify the strength of the relationship between effort discounting across domains (for model specifications, see Additional file 2). This analysis helped to ensure that we were accounting for task-specific variables, such as performance, that could influence the subjective value of cognitive effort across domains.

To extend the results of our pilot study, we then conducted a third test of our hypothesis, by additionally controlling for the influences of trait-level individual differences in working memory capacity and reward sensitivity when examining the association between the subjective value of cognitive effort across working memory and speech comprehension domains. For working memory capacity, we created a composite score, for which we summed the z-scores from the total score from each working memory measure (L-span, O-Span, Sym-Span). Reward sensitivity was calculated by summing the z-scores obtained in each reward sensitivity measure (BAS total score, GRAPES reward expectancy score, and the SPSRQ reward sensitivity score). The first step of the analysis was to examine the distributions and zero-order correlations involving these composite variables, as well as their association with the two subjective value estimates. Next, the two composite variables (working memory capacity, reward sensitivity) were included as covariates in a partial correlation analysis that used the cognitive effort discounting residual scores estimated for the second stage of analysis. We used the same uninformed prior distribution as detailed above, to measure the strength of the relationship between the subjective value (i.e., costs) of cognitive effort between working memory and speech comprehension domains, when controlling for the two individual difference measures.

This third stage of analysis was critical for determining whether the data provided support for a domain-general motivational construct that reflects the costs of cognitive effort, controlling for other relevant processes. We hypothesized that if this relationship existed, it would suggest that cognitive motivation can be indexed as a trait-like measure, such that measuring the subjective value of cognitive effort in one domain (e.g., working memory), would predict that an individual exhibits similar behavior in other cognitive domains**.** In contrast, if we found that the first hypothesis (a correlation between indifference points across the two effort discounting tasks) was confirmed, but the second hypothesis (a persistent correlation with added covariates) was disconfirmed, we would have concluded that cognitive motivation is domain specific. In other words, it is an individual’s working memory capacity and/or reward sensitivity that accounts for the relationship between the costs of cognitive effort across multiple cognitive (working memory, speech) domains. If the results from the third stage of analysis were inconclusive, we would decide that we could not draw firm conclusions regarding whether trait-level individual differences, such as working memory capacity and reward sensitivity, can account for the relationship between the subjective costs of cognitive effort across domains.

## Results

**Fig. 1:**
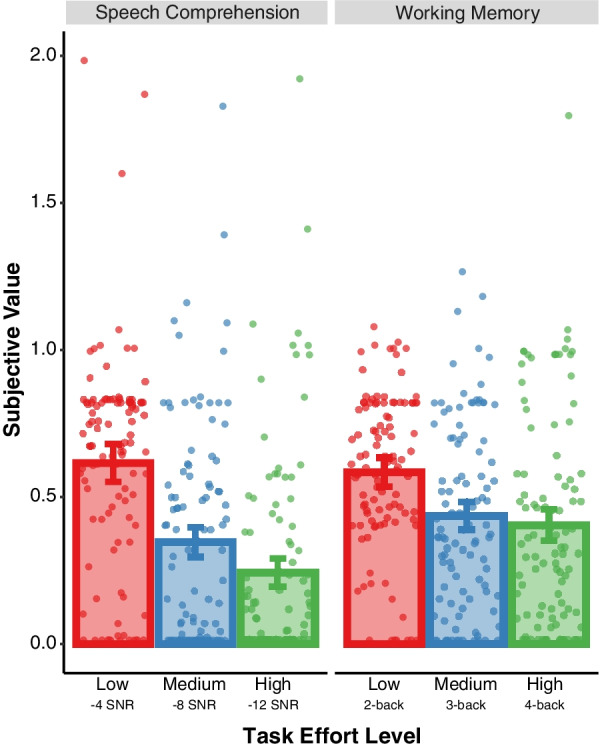
Effects of task load (low effort: 2-back, −4 dB SNR; medium effort: 3-back, −8 dB SNR; high effort: 4-back, −12 dB SNR) on subjective value estimates in working memory and speech comprehension domains. Data points represent subjective value estimates for each participant. Error bars represent 95% Confidence Intervals

Replicating the pattern of results observed in our pilot sample, we found that across both domains, participants discounted task load, such that more difficult levels of the task (e.g., blue; 3-Back, − 8 SNR) were discounted more, or had a lower subjective value, relative to easier task levels (e.g., red; 2-Back, − 4 SNR), *B* = − 0.09 [− 0.05, − 0.13], SD = 0.02 (Fig. 1). However, in contrast to the findings from the pilot sample, we found differences in discounting across domain, *B* = 0.12 [0.01, 0.24], SD = 0.06, with greater discounting observed in the speech comprehension task. Additionally, there was an interaction between task load and domain, *B* = − 0.10 [− 0.04, − 0.15], SD = 0.03; there was no difference in discounting between domains for low levels of effort (2-back, − 4SNR; *t*(103) = − 0.74 [− 0.12, 0.05], BF_10_ = 0.14), but greater discounting for speech comprehension was observed at medium (3-back, − 8SNR; *t*(103) = 2.19 [0.01, 0.17], BF_10_ = 1.06) and high levels of effort (4-back, − 12SNR; *t*(103) = 4.13 [0.08, 0.24], BF_10_ = 241.98). However, the Bayes Factor provided only strong support in favor of domain difference at the highest level of effort. Self-reported ratings of mental demand, effort, and frustration provided further support regarding the costs of cognitive effort in each domain. There was a main effect of task load across ratings of mental demand *B* = 3.69 [3.04, 4.39], SD = 0.35, effort *B* = 2.52 [1.89, 3.16], SD = 0.32, and frustration *B* = 1.04 [0.24, 1.83], SD = 0.41. This suggests that as task load level increased, subjective ratings of mental demand, effort, and frustration increased in each domain. Additional analyses of self-reported mental demand, effort, and frustration are summarized in Additional file 2.

**Fig. 2:**
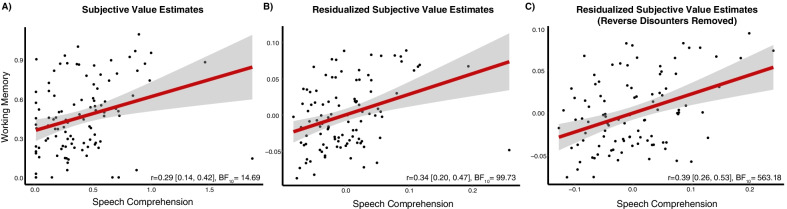
**a** Zero-order correlation of average subjective value estimates across working memory and speech domains, including all participants. **b** Correlation of residualized average subjective value estimates across working memory and speech domains, including all participants, but after controlling for task load and individual differences in task performance, working memory capacity, and reward sensitivity. **c** Correlation of residualized average subjective value estimates across working memory and speech domains, after excluding participants who had an average subjective value > 1, and also controlling for task load and individual differences in task performance, working memory capacity, and reward sensitivity

Our core hypothesis is that cognitive motivation is a stable, trait-like, construct that contributes to an individual’s cognitive effort costs. Our first test of this hypothesis was to examine the zero-order correlation between participant’s average subjective estimates in the working memory and speech comprehension domains. Replicating the results from our pilot sample, we found a positive relationship in COG-ED subjective value estimates across the two domains, *r* = 0.29 [0.14, 0.42], BF_10_ = 14.69 (Fig. 2a). In other words, participants who exhibited a low subjective value of cognitive effort (e.g., find engaging in cognitive effort to be costlier) in the working memory domain also tended to have a low subjective value of cognitive effort in the speech comprehension domain.

The second test of our hypothesis was to examine this association after first statistically controlling task load and individual variation in task performance within each domain. We found that task load was a significant predictor of subjective value in both the working memory, *B* = − 0.09 [− 0.12, − 0.06], SD = 0.01, *R*^2^ = 0.640, and speech comprehension domains, *B* = − 0.19 [− 0.22, − 0.16], SD = 0.02, *R*^2^ = 0.652. Furthermore, after adding *N*-back task performance variables to the multilevel model predicting subjective value estimates, we found that in addition to task load, *B* = − 0.07 [− 0.10, − 0.04], SD = 0.01, hit rate also explained variance in subjective value, *B* = 0.20 [0.09, 0.31], SD = 0.06, whereas correct rejection rate, *B* = − 0.05 [− 0.22, 0.13], SD = 0.09, and mean reaction time, *B* = 0.00 [− 0.00, 0.00], SD = 0.00, did not, *R*^2^ = 0.654. In the speech comprehension domain, only task load, *B* = − 0.12 [− 0.19, − 0.04], SD = 0.04, explained variance in subjective value estimates, whereas intelligibility did not, *B* = 0.00 [− 0.00, 0.00], SD = 0.00, *R*^2^ = 0.658. After extracting the residual variation unaccounted for by task load and performance, we found that the relationship between the costs of cognitive effort in working memory and speech comprehension domains still remained highly reliable, and even increased slightly *r* = 0.31 [0.17, 0.45], BF_10_ = 34.74.

The third and most critical test of our hypothesis was to statistically control for individual differences in working memory capacity and reward sensitivity, in addition to task load and performance. We first verified that these two composite measures were normally distributed and were not strongly associated with each other, *r* = − 0.14 [− 0.29, 0.02], BF_10_ = 0.386 (see Additional file 2). There was no relationship between the working memory capacity composite and averaged subjective value estimates in the working memory, *r* = 0.12 [− 0.03, 0.28], BF_10_ = 0.347, or speech comprehension domains, *r* = − 0.14 [− 0.29, 0.01], BF_10_ = 0.451. Likewise, there was no support for the relationship between the reward sensitivity composite and the averaged subjective value estimates in either the working memory, *r* = 0.13 [− 0.03, 0.27], BF_10_ = 0.365, or speech comprehension domain, *r* = − 0.09 [− 0.24, 0.06], BF_10_ = 0.231. Most importantly, we still observed a positive association between the costs of cognitive effort across domains (that were additionally residualized from task performance) after additionally controlling for these two individual differences measures, *r* = 0.34 [0.20, 0.47], BF_10_ = 99.73 (Fig. 2b). This suggests that participants’ discounting behavior reflects stable individual differences in cognitive motivation.

As an additional follow-up, when we removed all participants who had an average subjective value estimate > 1 (i.e., participants who almost always chose the high-effort option), the same pattern of results was still present; i.e., a positive relationship between effort discounting across working memory and speech comprehension domains, *r* = 0.31 [0.17, 0.45], BF_10_ = 21.91. This value further increased after controlling for task load and performance, *r* = 0.37 [0.23, 0.50], BF_10_ = 230.53, and individual differences in working memory capacity and reward sensitivity, *r* = 0.39 [0.26, 0.53], BF_10_ = 563.18 (see Additional file 2 for more information). Indeed, if anything, the exclusion of potential outlier participants and statistical control for potential covariates served to strengthen the association between the two estimates of cognitive motivation (see Fig. 2c).

### Exploratory analyses

To test for the associations between behavioral and self-reported measures of cognitive motivation, we correlated each participant’s average subjective value estimates in each domain with their score on the NCS. Confirming the results from our pilot sample, we did not find conclusive evidence for a relationship between self-reported and behavioral measures of cognitive motivation. Correlations between NCS and the working memory COG-ED (*r* = − 0.002 [− 0.15, 0.16], BF_10_ = 0.150), speech comprehension COG-ED (*r* = 0.12 [− 0.02, 0.28], BF_10_ = 0.364), and the composite COG-ED score (*r* = 0.08 [− 0.08, 0.23], BF_10_ = 0.211) were not robust. Furthermore, even when correlating the residualized estimates of subjective value, controlling for task load and individual differences in task performance, working memory capacity, and reward sensitivity, we did not observe a relationship between NCS and the working memory COG-ED (*r* = − 0.03 [− 0.18, 0.13], BF_10_ = 0.158) or the speech comprehension COG-ED (*r* = 0.12 [− 0.04, 0.26], BF_10_ = 0.303).

## Discussion

Cognitive motivation is an important construct involved in decision-making processes. However, existing studies have largely assessed individual differences in cognitive motivation using retrospective global self-report measures, which provide a limited view of the construct. Here, we used the COG-ED paradigm, which makes use of behavioral economics methodology to frame individual differences in cognitive motivation as the trade-off between the costs and benefits of engaging in cognitive effort. To test our central hypothesis regarding the domain generality of cognitive motivation, we used the COG-ED to assess participants across two distinct cognitive domains (working memory, speech comprehension). In addition, we collected individual difference measures of working memory capacity and reward sensitivity, which allowed us to control for these processes when measuring the costs of cognitive effort across working memory and speech comprehension domains. We replicated previous work using the COG-ED, which showed that with increasing task load in both working memory (Westbrook et al., 2013) and speech comprehension domains (McLaughlin et al., 2021), participants discount higher effort options more than lower effort options. Critically, we extended these findings to show that participants who exhibited the highest costs of cognitive effort in one domain also tended to show this same pattern of behavior in the other domain. Interestingly, we also found that when we controlled for both task load and individual differences in task performance, working memory capacity, and reward sensitivity, there was even stronger evidence for the relationship between the costs of cognitive effort within individuals across working memory and speech comprehension domains, which might suggest that these other factors suppress the domain-general relationship between discounting across cognitive domains. Taken together, these results provide support for the hypothesis that cognitive motivation operates in a domain-general fashion within individuals. Moreover, we suggest that this construct of cognitive motivation contributes to an individual’s decision to engage or not engage with cognitively demanding activities.

A key takeaway from our study is that behavioral decision-making paradigms are a powerful tool that can be used to measure individual differences in cognitive motivation across distinct cognitive domains. By isolating an individual’s costs of cognitive effort using revealed preferences with the COG-ED, we can precisely examine the influence of contextual factors, such as task load and domain, on their decision-making behavior. In other words, the experimental indices from the COG-ED (i.e., the subjective value of cognitive effort) provide us with a unique approach to assess the domain generality of cognitive motivation within individuals that is independent of the retrospective, self-report measures commonly used to assess this construct (e.g., NCS). Indeed, our findings parallel other recent work using the DST, an effort-related decision-making paradigm (Strobel et al., 2020). Strobel et al (2020) also showed that across two distinct cognitive domains, a trait-like cognitive motivational variable was identified when using decision-making paradigms to measure an individual’s willingness to engage in cognitive effort, even when controlling for other variables, such as cognitive ability. When taken together, our results and that of Strobel et al (2020) highlight the utility of behavioral decision-making paradigms for quantifying individual differences in cognitive motivation across multiple cognitive domains.

Furthermore, our finding of a stable, trait-like, cognitive motivational construct that contributes to an individual’s cognitive effort costs is broadly consistent with neural frameworks that are thought to support cognitive motivation. There is a growing body of research that has aimed to assess the neurobiological substrates of cognitive motivation (Lopez-Gamundi et al., 2021; Westbrook et al., 2021). This work suggests the presence of a domain-general network with core nodes in the ventromedial prefrontal cortex and ventral striatum, which indexes individual differences in the value of cognitive effort (Lopez-Gamundi et al., 2021). For example, results from one study showed that activity in the ventral striatum during cognitive effort discounting in younger adults reflects individual differences in the costs of cognitive effort (i.e., cognitive motivation) (Westbrook et al., 2019). Moreover, the role of the neurotransmitter dopamine has long been hypothesized as playing a critical role in cognitive motivation (Aarts et al., 2011; Westbrook & Braver, 2016). More recent work has demonstrated that younger adults with low dopamine synthesis capacity show reduced cognitive motivation (i.e., higher costs of cognitive effort) relative to those with higher levels at baseline (Westbrook et al., 2020). Conversely, administration of a dopamine agonist (methylphenidate) was found to boost cognitive motivation in participants with low baseline synthesis capacity, while decreases in cognitive motivation were found in participants with high baseline synthesis capacity after drug administration (Hofmans et al., 2020). These results suggest that cognitive motivation relies in part on the delicate balance of the striatal dopamine system when weighing the costs and benefits of engaging in cognitive effort. However, to date, there has been no work that has aimed to assess the neural mechanisms of cognitive motivation across distinct cognitive domains within participants. Such work could easily be extended from the current study, by assessing whether activity in the ventromedial prefrontal cortex and ventral striatum show a similar activity profile when participants are asked to make decisions about engaging in cognitive effort across distinct cognitive domains and whether individual differences in dopamine show similar linkages between cognitive motivation across different cognitive domains. Such findings, if obtained, would provide an even stronger test of the domain generality of cognitive motivation, in terms of underlying neural mechanisms.

It is important to note that we did not find support for a relationship between self-reported and behavioral indices of cognitive motivation. There was no correlation between NCS and the average subjective value of cognitive effort in either working memory or speech comprehension domains, even when controlling for individual differences in task performance, working memory capacity, and reward sensitivity. Although this finding lies in contrast to our initial predictions (and previous findings with the COG-ED; (Kramer et al., 2021; Westbrook et al., 2013), it highlights a potentially important distinction between the self-reported and behavioral measures of cognitive motivation used in this study. Put another way, by defining and quantifying the impact of contextual factors (e.g., task load, cognitive domain) on cognitive motivation using the COG-ED, we diverge from the global self-report measures used to study individual differences in cognitive motivation. In particular, whereas global self-report measures (e.g., NCS) ask participants to retrospectively report, or generalize about, their experiences and motivations, experimental paradigms, like the COG-ED, tightly control the decision-making context and quantify participants’ behaviors from a narrow range of cognitive tasks to form an index of cognitive motivation. Thus, it is possible that both types of assessments measure cognitive motivation, but they do not index the same properties of the construct. Indeed, others have shown that there is often a weak correspondence between self-reported and behavioral indices of the same construct and suggest that this occurs in part because the two types of measures tap into distinct response processes (Dang et al., 2020). Recent work using both self-reported and behavioral measures of cognitive motivation also shows this pattern of results, wherein self-reported and behavioral measures both showed trait variance but did not have any relation to one another (Bolenz et al., 2020; Strobel et al., 2020). However, to our knowledge, no studies have examined the trait construct of cognitive motivation using multiple behavioral paradigms (e.g., COG-ED and DST) in addition to self-report measures to test for the strength of the association between the self-reported and behavioral measures.

## Conclusion

Cognitive motivation is an important construct used to understand individual differences in many facets of daily life, including the ability to seek out and scrutinize information and achieve academic and career success. In the current study, we found that participant’s choices on a behavioral decision-making paradigm (COG-ED) were associated across two distinct cognitive domains (e.g., working memory, speech comprehension), consistent with a stable, trait-like individual differences construct of cognitive motivation. Importantly, we observed this trait-like tendency to engage in cognitive effort even after controlling for task load and individual differences in task performance, working memory capacity, and reward sensitivity. These findings suggest that in healthy, young adult participants cognitive motivation operates in a domain-general fashion, and as such can be successfully measured using behavioral paradigms, such as the COG-ED. Future work can employ these methods to more rigorously study the altered cognitive effort costs and decision making that may be occurring in other populations where changes in cognitive motivation have been observed, such as in individuals with neuropsychiatric disorders (e.g., depression, schizophrenia), as well as in both healthy and pathological aging.

## Supplementary Information

**Additional file 1.** Pilot data description and analyses.

**Additional file 2.** Supplementary material for Main Text.

## Data Availability

All relevant experimental scripts, data, code, and analyses are in an online repository on the Open Science Framework: https://osf.io/9t6q7/.
