# Supplementary material for: Domain-general cognitive motivation: Evidence from economic decision-making – Final Registered Report
Source: Cogn Res Princ Implic. 2022 Mar 18;7:23. doi: 10.1186/s41235-022-00363-z (PMC8931160; doi:10.1186/s41235-022-00363-z)
Supplement: Supplementary file 1 — Additional file 1. Pilot data description and analyses. [file 41235_2022_363_MOESM1_ESM.pdf]

Supplement: Pilot Findings

Pilot Data

Participants
Thirty-two participants were recruited from the Washington University Psychological & Brain Sciences Department subject pool. All participants provided written informed consent and were given payment of $10/hour in addition to task-based earnings. One participant ended the experimental session prior to completion and was excluded from analysis. Consequently, the final sample consisted of thirty-one participants (21 females; ages 18-23 years; M = 19.84 SD = 1.39). All participants were native English speakers, reported no current or previous history of neurological trauma, seizures, or mental illness, and no use of psychotropic medications. The Washington University Human Research Protections Office approved all experimental procedures.

Materials & Methods
All stimuli presented in the speech-in-noise task were recordings of a female, native speaker of American English, reading sentences with four keywords each (e.g., "the *thirsty dog drank* the *water*")[34]. Sentences were presented in speech-shaped noise that was created to have the same long-term average spectrum of the sound files. Stimuli were mixed with Praat version 6.0.16, and presented at signal-to-noise ratios (SNRs) of 0, -4, -8, or -12 dB.

All participants performed a cognitive effort discounting paradigm (COG-ED) following the same basic structure as prior work (Westbrook et al., 2013). The procedure of the familiarization phase(s) and discounting procedures used in this experiment are nearly identical to the procedures described in the manuscript.

As a brief overview, participants completed familiarization and discounting procedures within each cognitive domain. The familiarization phase consisted of practice performing variously demanding levels of either a working memory (N-Back) or speech-in-noise task, with task order counterbalanced across participants. During the working memory task, participants completed two runs of each level of the task (N=1-4) in ascending order of difficulty. During the speech-in-noise task, participants were presented with sentences with varying levels of noise (i.e., 0 dB SNR, -12 dB SNR) through headphones and were instructed to repeat the sentence back to the experimenter, who was in the room recording the responses. Participants were instructed to guess if they were unsure of any words in a sentence. Like the working memory task, participants completed task blocks in order of difficulty, from easiest (i.e., 0 SNR) to hardest (i.e., -12 SNR).

Following each run of the familiarization task (e.g., completing the 1-Back or 0 SNR task), participants completed self-reported ratings of the mental demand, physical demand, temporal demand, effort, frustration, and performance from the preceding task block using the NASA Task Load Index[28]. Participants provided their responses using a visual analog scale ranging from 0 (very low) - 100 (very high).

After the familiarization phase participants made repeated choices about whether to repeat performance of a higher load-level of the task (e.g. 4-back, -12 SNR) for a fixed high reward ($2, $3, $4), or instead perform the easiest load level (1-back, 0 SNR) for a variable, lower reward (i.e., decision-making phase). Participants completed a total of 45 decision trials in each domain and were informed that one of their choices would be used to determine task-based compensation and that they would be asked to repeat the task they chose, for the amount of money offered (i.e., $2 for the “red” task). Furthermore, participants were informed that task-

based compensation was not based on performance from the familiarization phase, but rather, they were told that to successfully earn the money for repeating the chosen task, they needed to maintain their effort from the familiarization block when repeating the task block. After completing all task blocks in each domain, participants were asked to complete a post-task questionnaire to assess how much their choices during the discounting phase were based on the difficulty, effort, or monetary reward associated with the task.

Analysis

Bayesian linear mixed effect models were conducted in the package *brms* (version 2.12.0), R version 3.62 (RRID:SCR_001905) to estimate the effects of task load and domain (i.e., working memory, speech comprehension) on both participants' discounting behaviors and their self-reported ratings of mental demand, effort, and frustration. In all models, task, domain, and their interaction were entered as fixed effects, with a random effect of intercept. Further, we used default prior distributions in *brms* for each of the fixed effects (i.e., flat prior; central t-distribution, df=3) and number of iterations (4000) for each of these models, providing an estimate equivalent to maximum likelihood approaches used in multilevel modeling (e.g., using the package *lme4*). In the reported results, we provide the beta estimate (i.e., mean of the posterior distribution), the 95% credible intervals, and the standard deviation of the posterior distribution (i.e., error).

To test for the strength of the association between the costs of cognitive effort across working memory and speech comprehension domains, we performed a correlation test using the *BayesFactor* (version 0.9.12-4.2) package in R. For this analysis, we used an uninformed prior, Cauchy distribution ($\mu = 0$, $r = \sqrt{2}/2$) and report the correlation value as the median of the posterior distribution, in addition to the 95% credible intervals, and the Bayes factor, contrasting the

strength of the experimental model (i.e., correlation between effort costs across domains), relative to the null hypothesis, (i.e., no correlation between effort costs across domains).

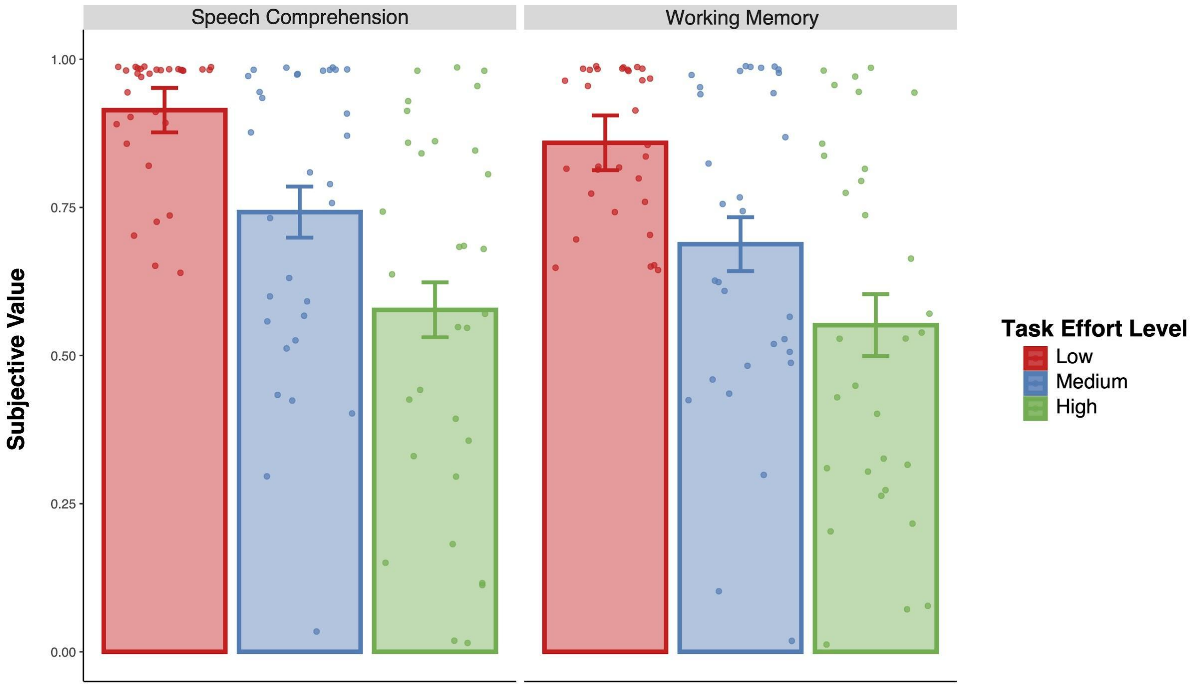

Figure S1. Effects of task load (low effort: 2-back, -4 SNR; medium effort: 3-back, -8S NR; high effort: 4-back, -12 SNR) on subjective value estimates in working memory and speech comprehension domains. Error bars represent 95% CIs.

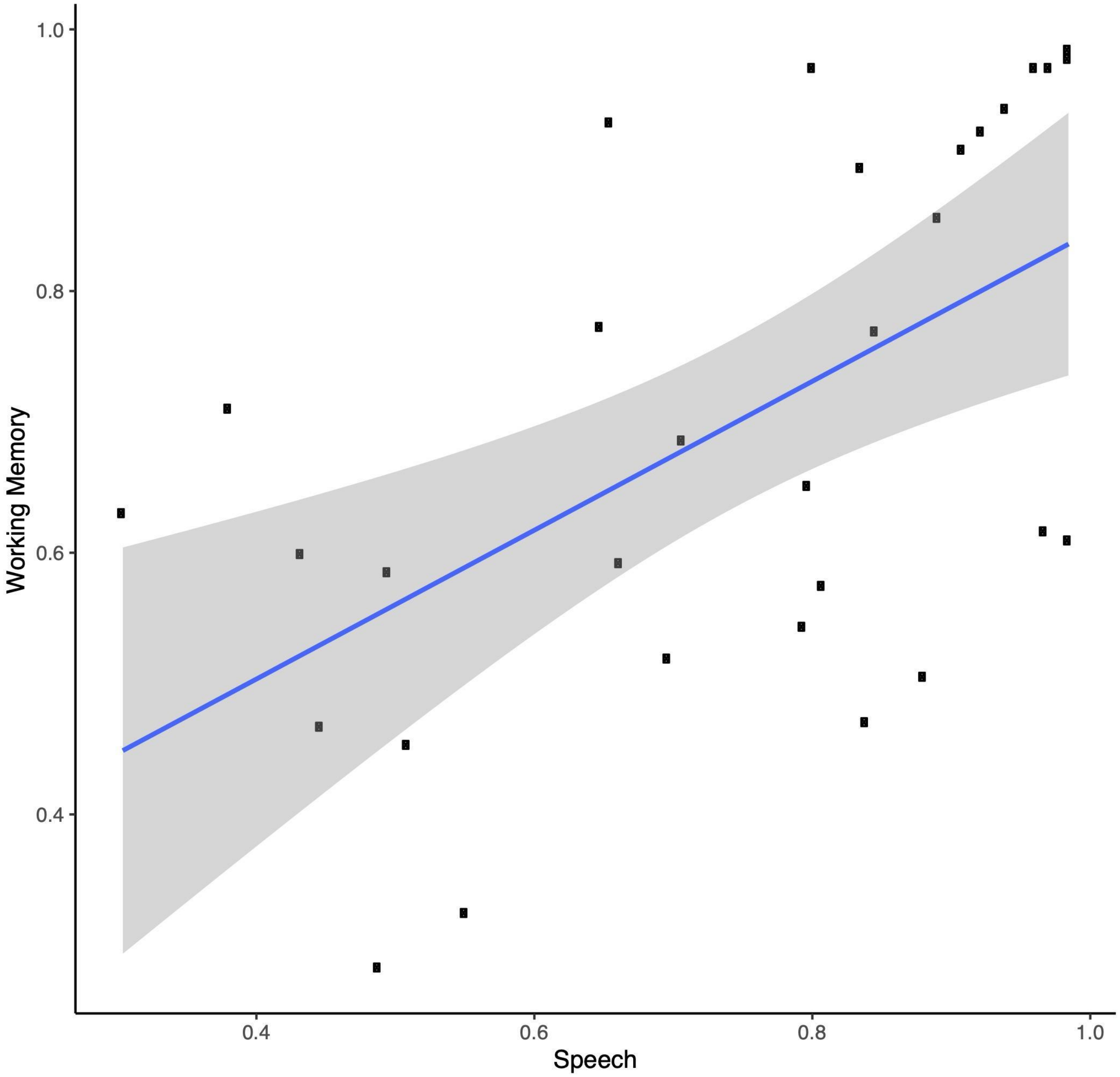

Figure S2. Correlation of average subjective value estimates across working memory and speech domains.

Power Analysis

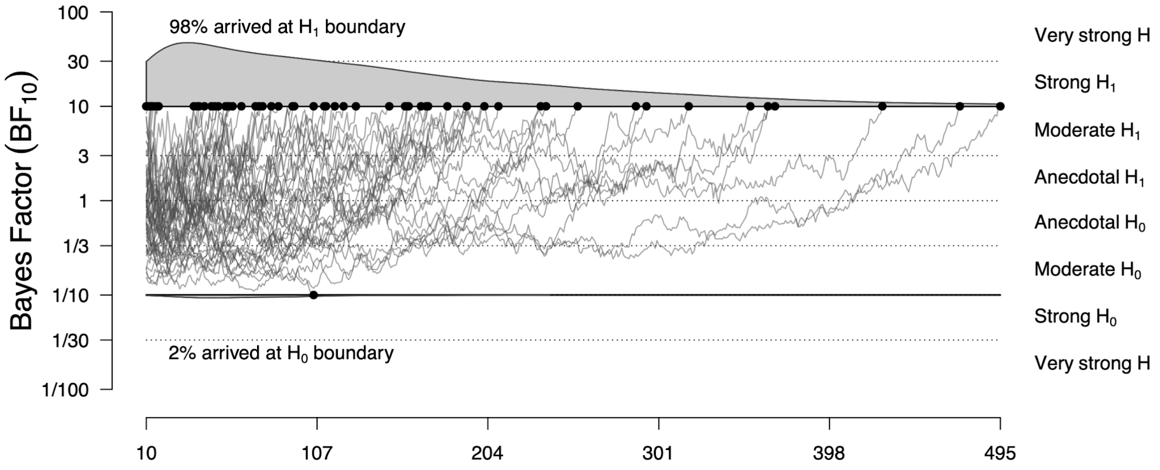

Figure S3. Summary plot of simulations used to approximate sample size (x-axis) using sequential BFDA. [results generated with the BFDA package]

Additional Questionnaires

In addition to collecting the self-report measures described in the manuscript, we collected a measure of subjective hearing ability in a subset of participants (Speech, Spatial and Qualities of Hearing Scale; SSQ). Further, we asked participants to complete a post-task questionnaire after each discounting task, which quantified their motivations for their choices during the decision-making task (i.e., influence of monetary reward available, task difficulty). In addition, all participants answered questions about type of device they used to complete the tasks in the speech domain (e.g., headphones, speakers) to control for differences in presentation method.
