# Supplementary material for: Domain-general cognitive motivation: Evidence from economic decision-making – Final Registered Report
Source: Cogn Res Princ Implic. 2022 Mar 18;7:23. doi: 10.1186/s41235-022-00363-z (PMC8931160; doi:10.1186/s41235-022-00363-z)

Supplement for 'Domain-general cognitive motivation: evidence from economic decision-making'

Jennifer L. Crawford

10/1/2021

Supplemental Information & Analyses for Stage II Manuscript

The following code and reported analyses provide additional information beyond what is presented in the code accompanying the main text findings (MDD_online_share.Rmd). Below, you will find all pertinent packages and paths to the data used to conduct these supplemental analyses.

```
rm(list=ls())

#Packages
library(brms); library(bayestestR); library(BayesFactor); library(correlation
); library(knitr);
library(RColorBrewer); library(tidyverse);
source("summarySEwithin2.R")

#Package versions used to run the analyses reported in the manuscript
##R version 4.1.0
##BayesFactor_0.9.12-4.2, bayestestR_0.10.5, brms_2.16.1, correlation_0.6.1,
knitr_1.33,
##RColorBrewer_1.1-2, tidyverse_1.3.1

#Create data directories
#Cog-ED
coged.wm.path<-"https://raw.githubusercontent.com/jlcrawford/MDD/master/Data/
Online/Discounting/MDD_WMCogED_share.csv"
coged.wm.full.path <-"https://raw.githubusercontent.com/jlcrawford/MDD/master
/Data/Online/Discounting/MDD_WMCogED_full_share.csv"
coged.speech.path<-"https://raw.githubusercontent.com/jlcrawford/MDD/master/D
ata/Online/Discounting/MDD_SpeechCogED_share.csv"
#Individual Difference Questionnaires
NCS.path <- "https://raw.githubusercontent.com/jlcrawford/MDD/master/Data/Onl
ine/Individual-Difference-Questionnaires/MDD_NCS_share.csv"
SPSRQ.path <- "https://raw.githubusercontent.com/jlcrawford/MDD/master/Data/O
nline/Individual-Difference-Questionnaires/MDD_SPSRQ_share.csv"
BISBAS.path <- "https://raw.githubusercontent.com/jlcrawford/MDD/master/Data/
Online/Individual-Difference-Questionnaires/MDD_BISBAS_share.csv"
GRAPES.path <- "https://raw.githubusercontent.com/jlcrawford/MDD/master/Data/
Online/Individual-Difference-Questionnaires/MDD_GRAPES_share.csv"
```

```
#Working Memory Capacity
LSpan.path <- "https://raw.githubusercontent.com/jlcrawford/MDD/master/Data/O
nline/Working-Memory-Capacity/MDD_LSpan_share.csv"
OSpan.path <- "https://raw.githubusercontent.com/jlcrawford/MDD/master/Data/O
nline/Working-Memory-Capacity/MDD_OSpan_share.csv"
SymmSpan.path <- "https://raw.githubusercontent.com/jlcrawford/MDD/master/Dat
a/Online/Working-Memory-Capacity/MDD_SSpan_share.csv"
#complete subject list
subjects.path <- "https://raw.githubusercontent.com/jlcrawford/MDD/master/Dat
a/Online/Discounting/subjects_online_MDD.csv"
#Make data frames for Cog-ED and demographics info
coged.wm<- read.csv(coged.wm.path, header = T)
coged.wm.full <- read.csv(coged.wm.full.path, header = T)
coged.speech<- read.csv(coged.speech.path, header = T)
#Make data frames for individual difference questionnaires
NCS <- read.csv(NCS.path, header = T)
SPSRQ <- read.csv(SPSRQ.path, header = T)
BISBAS <- read.csv(BISBAS.path, header = T)
GRAPES <- read.csv(GRAPES.path, header = T)
#Make data frames for working memory capacity tasks
LSpan <- read.csv(LSpan.path, header = T)
OSpan <- read.csv(OSpan.path, header = T)
SymmSpan <- read.csv(SymmSpan.path, header = T)
#Make data frame for usable subject info (i.e., subjects who have completed a
ll tasks and questionnaires across both visits)
Subjects <- read.csv(subjects.path, header = F)
colnames(Subjects) <- "subjectid"
```

Removing Participants who have an average SV >1

As an additional follow-up, when we removed all participants who had an average subjective value estimate >1 (i.e., participants who almost always chose the high-effort option), the same pattern of results holds; there is a positive relationship between effort discounting across working memory and speech comprehension domains, r = 0.31 [0.16, 0.45], BF_{10}= 21.91, which remains after controlling for task difficulty and performance, r = 0.37 [0.23, 0.51], BF_{10}= 230.53, and individual differences in working memory capacity and reward sensitivity, r = 0.39 [0.26, 0.53], BF_{10}= 563.18.

Zero-order correlation between working memory and speech subjective value estimates

Five participants showed patterns of reverse discounting and were removed from the full dataset to conduct the following analyses.

99 participants inlcuded in analyses

```
#Cog-ED Data
#clean data frame(s) with Cog-ED subjective value (SV) estimates and transfor
m data so that SV
#estimates are equivalent across both domains (i.e., speech, WM)
```

```
##working memory
d.coged.wm <- coged.wm %>% select(subjectid,completed,fixedAmount_N2_1,
                            fixedAmount_N2_2,fixedAmount_N2_3,
                            fixedAmount_N3_1,fixedAmount_N3_2,
                            fixedAmount_N3_3,fixedAmount_N4_1,
                            fixedAmount_N4_2,fixedAmount_N4_3,
                            IP12_1,IP12_2,IP12_3,IP13_1,IP13_2,IP13_3,
                            IP14_1,IP14_2,IP14_3) %>%
  filter(completed == 1) %>%
  group_by(subjectid) %>%
  mutate(Domain = "WM",
         domainCode = 0,
         SV2_1 = ifelse(fixedAmount_N2_1 == "X", IP12_1/2, ((2-IP12_1)/2)+1),
         SV2_2 = ifelse(fixedAmount_N2_2 == "X", IP12_2/3, ((3-IP12_2)/3)+1),
         SV2_3 = ifelse(fixedAmount_N2_3 == "X", IP12_1/4, ((4-IP12_3)/4)+1),
         SV3_1 = ifelse(fixedAmount_N3_1 == "X", IP13_1/2, ((2-IP13_1)/2)+1),
         SV3_2 = ifelse(fixedAmount_N3_2 == "X", IP13_2/3, ((3-IP13_2)/3)+1),
         SV3_3 = ifelse(fixedAmount_N3_3 == "X", IP13_1/4, ((4-IP13_3)/4)+1),
         SV4_1 = ifelse(fixedAmount_N4_1 == "X", IP14_1/2, ((2-IP14_1)/2)+1),
         SV4_2 = ifelse(fixedAmount_N4_2 == "X", IP14_2/3, ((3-IP14_2)/3)+1),
         SV4_3 = ifelse(fixedAmount_N4_3 == "X", IP14_3/4, ((4-IP14_3)/4)+1),
         SV_red = (SV2_1 + SV2_2 + SV2_3)/3,
         SV_blue = (SV3_1 + SV3_2 + SV3_3)/3,
         SV_purple = (SV4_1 + SV4_2 + SV4_3)/3)

##speech comprehension
d.coged.speech <- coged.speech %>%
  select(subjectid,completed,fixedAmount_N2_1,fixedAmount_N2_2,fixedAmount_N2
_3,
                            fixedAmount_N3_1,fixedAmount_N3_2,fixedAmount_N3_
3,
                            fixedAmount_N4_1,fixedAmount_N4_2,fixedAmount_N4_
3,
                            IP12_1,IP12_2,IP12_3,IP13_1,IP13_2,IP13_3,IP14_1,
IP14_2,IP14_3) %>%
  filter(completed == 1) %>%
  group_by(subjectid) %>%
  mutate(Domain = "Speech",
         domainCode = 1,
         SV2_1 = ifelse(fixedAmount_N2_1 == "X", IP12_1/2, ((2-IP12_1)/2)+1),
         SV2_2 = ifelse(fixedAmount_N2_2 == "X", IP12_2/3, ((3-IP12_2)/3)+1),
         SV2_3 = ifelse(fixedAmount_N2_3 == "X", IP12_1/4, ((4-IP12_3)/4)+1),
         SV3_1 = ifelse(fixedAmount_N3_1 == "X", IP13_1/2, ((2-IP13_1)/2)+1),
         SV3_2 = ifelse(fixedAmount_N3_2 == "X", IP13_2/3, ((3-IP13_2)/3)+1),
         SV3_3 = ifelse(fixedAmount_N3_3 == "X", IP13_1/4, ((4-IP13_3)/4)+1),
         SV4_1 = ifelse(fixedAmount_N4_1 == "X", IP14_1/2, ((2-IP14_1)/2)+1),
         SV4_2 = ifelse(fixedAmount_N4_2 == "X", IP14_2/3, ((3-IP14_2)/3)+1),
         SV4_3 = ifelse(fixedAmount_N4_3 == "X", IP14_3/4, ((4-IP14_3)/4)+1),
         SV_red = (SV2_1 + SV2_2 + SV2_3)/3,
         SV_blue = (SV3_1 + SV3_2 + SV3_3)/3,
```

```
        SV_purple = (SV4_1 + SV4_2 + SV4_3)/3)
#Merge WM and Speech Cog-ED data frames
coged.merged <- rbind(d.coged.wm, d.coged.speech)

#Filter out subjects who have not completed all tasks in the protocol
coged.merged <-inner_join(Subjects, coged.merged)

#Add dummy variables (task, domain) for multilevel models
d.coged.SV <- coged.merged %>% select(subjectid,Domain,domainCode, SV_red, SV
_blue, SV_purple) %>%
  pivot_longer(names_to = "tmp", values_to = "SV", -c(subjectid,Domain,domain
Code)) %>%
  separate(col = tmp, into=c(NA,"Task"), sep = "_") %>%
  mutate(taskCode = factor(Task, levels=c("red","blue","purple"), labels=c(-1
,0,1)))
d.coged.SV$taskCode <- as.numeric(d.coged.SV$taskCode)
d.coged.SV$domainCode <- as.numeric(d.coged.SV$domainCode)

#Correlating Average SV (within-subj) across domains
average.SV.outliers <- coged.merged %>% select(subjectid, Domain, SV_red, SV_
blue, SV_purple) %>%
  group_by(subjectid, Domain) %>%
  dplyr::summarise(SV_avg = (SV_red + SV_blue + SV_purple)/3) %>%
  pivot_wider(values_from = "SV_avg", names_from = "Domain") %>%
  filter(Speech < 1) %>% filter(WM < 1)
```

There is a positive relationship between effort discounting across working memory and speech comprehension domains, r = 0.31 [0.16, 0.45], BF_{10}= 21.91.

```
#Testing for correlation between cognitive effort discounting across working
memory & speech domains
CogED.cor.adj <- cor_test(data = average.SV.outliers, x = "Speech", y = "WM",
                          bayesian = TRUE, bayesian_prior = 0.707107)
#Summarize Bayes Factor from correlation
CogED.cor.adj
```

```
## Parameter1 | Parameter2 |  rho |       95% CI |      pd | % in ROPE |
Prior |      BF
## ---------------------------------------------------------------------------
-------------------------
## Speech     |         WM | 0.31 | [0.17, 0.45] | 100%*** |     1.32% | Beta
(1.41 +- 1.41) | 21.91**
##
## Observations: 99
```

```
#Plot of correlation between working memory & speech comprehension domains
fig.outlier.rm <- ggplot(average.SV.outliers, aes(Speech, WM)) +
   theme(plot.title = element_text(hjust = 0.5), panel.grid.major = element_b
```

```
lank(),
        panel.grid.minor = element_blank(), panel.background = element_blank
(),
        axis.line = element_line(colour = "black")) +
  geom_point() + geom_smooth(method=lm) +ggtitle("Reverse Discounters Removed
") +
    xlab("Speech Comprehension") + ylab("Working Memory")
fig.outlier.rm
```

Below, is the zero-order correlation between discounting across working memory and speech comprehension domains after removing participants who had an average subjective value > 1.

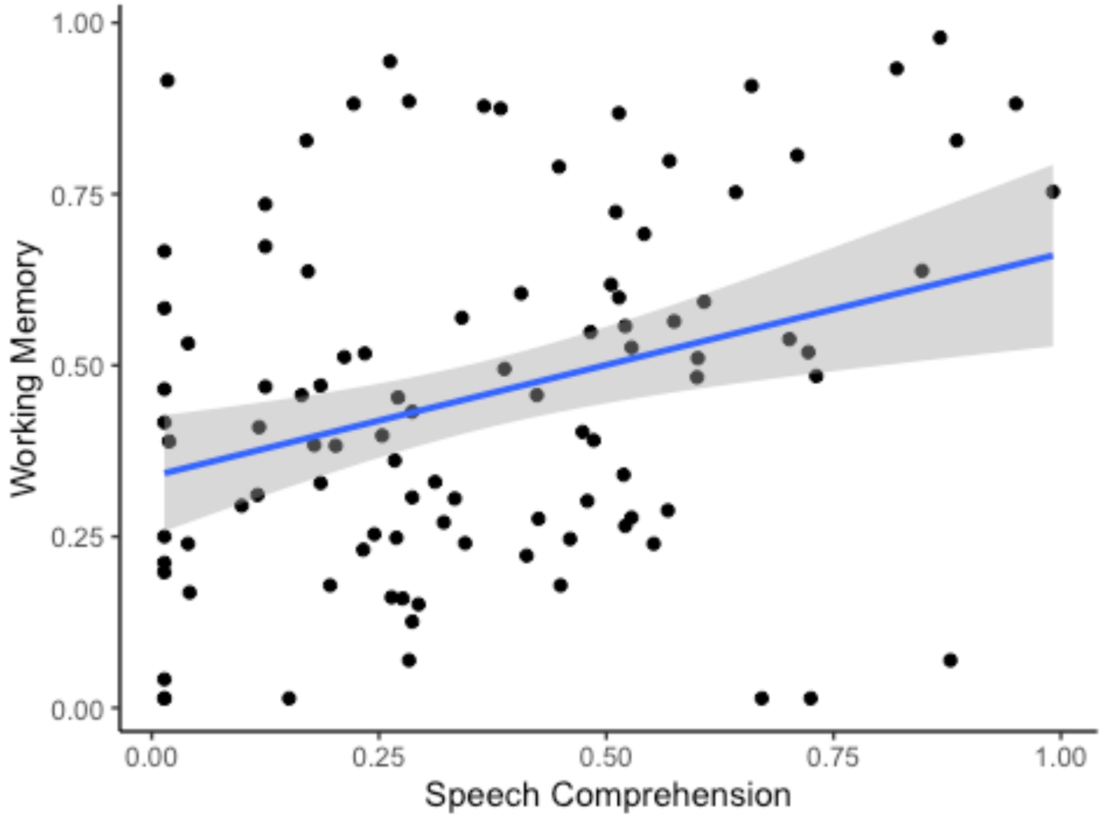

Controlling for task level and performance

The analyses below first summarize performance during the familiarization phase in both domains. We then use multilevel models to control for these performance variables, in addition to task load, and extract the residuals, which are then used in the correlation between discounting across working memory and speech comprehension domains.

Familiarization Phase Performance

Speech

```
performance.speech <- coged.speech %>% select(subjectid, completed, percentCo
rrect_N1,
                                              percentCorrect_N2, percentCorre
```

```
ct_N3, percentCorrect_N4) %>%
   group_by(subjectid) %>% filter(completed == 1) %>% select(-completed) %>%
  pivot_longer(names_to = "level", values_to = "performance", -c(subjectid))
%>%
  separate(col = level, into=c(NA,"Task"), sep = "_") %>% inner_join(Subjects
)
performance.speech$task <- factor(performance.speech$Task, levels = c("N1","N
2","N3","N4"),
                                  labels = c("black","red","blue","purple"))

performance_sum <- summarySEwithin2(performance.speech, measurevar = "perform
ance",
                                    withinvars = c("Task"), idvar = "subjecti
d")
performance_sum$Task <- factor(performance_sum$Task, levels = c("N1","N2","N3
","N4"),
                               labels = c("0 SNR","-4 SNR","-8 SNR","-12 SNR"
))
```

Working Memory

```
performance.wm <- coged.wm %>% select(subjectid, completed, hitrate_N1, CRrat
e_N1,
                                      hitrate_N2, CRrate_N2, hitrate_N3, CRra
te_N3,
                                      hitrate_N4, CRrate_N4) %>%
   group_by(subjectid) %>% filter(completed == 1) %>% select(-completed) %>%
  pivot_longer(names_to = "level", values_to = "performance", -c(subjectid))
%>%
  separate(col = level, into=c("Metric","Task"), sep = "_") %>%
  pivot_wider(names_from = Metric, values_from = performance)  %>% inner_join
(Subjects)
performance.wm$task <- factor(performance.wm$Task, levels = c("N1","N2","N3",
"N4"),
                              labels = c("black","red","blue","purple"))

performance.sum.wm <- summarySEwithin2(performance.wm, measurevar = "hitrate"
,
                                       withinvars = c("Task"), idvar = "subje
ctid")
performance.sum.wm$Task <- factor(performance.sum.wm$Task, levels = c("N1","N
2","N3","N4"),
                                  labels = c("1-back","2-back","3-back","4-ba
ck"))

performance.wm.RT <- coged.wm.full %>% select(subjectid, blockcode, phase, re
sponse, latency) %>%
  filter(phase == 1) %>%
  rename(task = "blockcode") %>% filter(task != "ratingSummary") %>% filter(r
esponse != 0)
performance.wm.RT$task <- factor(performance.wm.RT$task, levels = c("1back","
```

```
2back","3back","4back"),
                                 labels = c("black","red","blue","purple"))

performance.wm.RT.sum <-inner_join(performance.wm.RT, Subjects) %>% group_by(
subjectid, task) %>%
  summarise(meanRT = mean(latency))

#Working Memory Cog-ED
#Summarize average performance on N-Back
d.coged.wm.clean <- d.coged.wm %>% select(subjectid, SV_red, SV_blue, SV_purp
le) %>%
  pivot_longer(names_to = "tmp", values_to = "SV", -subjectid) %>%
   separate(col = tmp, into=c(NA,"task"), sep = "_")

d.coged.wm.partial <- d.coged.wm.clean %>% group_by(subjectid,task) %>%
  summarise(meanSV = mean(SV)) %>%
  inner_join(performance.wm, by = c("subjectid","task")) %>%
  inner_join(performance.wm.RT.sum) %>%
  mutate(taskCode = factor(task, levels =c( "black","red","blue","purple"),
                           labels = c(-2,-1,0,1)),
         FArate = 1-CRrate,
         HR_z = scale(hitrate),
         FAR_z = scale(FArate),
         dPrime = HR_z - FAR_z)

d.coged.wm.partial$taskCode <- as.numeric(d.coged.wm.partial$taskCode)

#Speech Cog-ED
d.coged.speech.clean <- d.coged.speech %>% select(subjectid, SV_red, SV_blue,
SV_purple) %>%
  pivot_longer(names_to = "tmp", values_to = "SV", -subjectid) %>%
   separate(col = tmp, into=c(NA,"task"), sep = "_")

d.coged.speech.partial <- d.coged.speech.clean %>% group_by(subjectid,task) %
>%
  summarise(meanSV = mean(SV)) %>%
  inner_join(performance.speech, by = c("subjectid","task")) %>%
  mutate(taskCode = factor(task, levels =c("red","blue","purple"),
                           labels = c(-1,0,1)))
d.coged.speech.partial$taskCode <- as.numeric(d.coged.speech.partial$taskCode
)

#Working Memory Cog-ED
#Summarize average performance on N-Back
outlier.subjs <- average.SV.outliers$subjectid %>% as_tibble()
colnames(outlier.subjs) <- "subjectid"

d.coged.wm.partial.outlier <- inner_join(d.coged.wm.partial, outlier.subjs)

m.SV.wm.partial.outlier <- brm(data = d.coged.wm.partial.outlier, meanSV ~ ta
```

```
skCode +
                                 hitrate + CRrate + meanRT + (1 | subjectid),
                              file = "models/m.SV.wm.partial.outlier.rds")
summary(m.SV.wm.partial.outlier)
```

```
##  Family: gaussian
##   Links: mu = identity; sigma = identity
## Formula: meanSV ~ taskCode + hitrate + CRrate + meanRT + (1 | subjectid)
##    Data: d.coged.wm.partial.outlier (Number of observations: 297)
##   Draws: 4 chains, each with iter = 2000; warmup = 1000; thin = 1;
##          total post-warmup draws = 4000
##
## Group-Level Effects:
## ~subjectid (Number of levels: 99)
##               Estimate Est.Error l-95% CI u-95% CI Rhat Bulk_ESS Tail_ESS
## sd(Intercept)     0.23      0.02     0.20     0.28 1.00     1279     2301
##
## Population-Level Effects:
##           Estimate Est.Error l-95% CI u-95% CI Rhat Bulk_ESS Tail_ESS
## Intercept     0.58      0.13     0.32     0.84 1.00     2274     2871
## taskCode     -0.08      0.01    -0.11    -0.05 1.00     4691     3035
## hitrate       0.20      0.06     0.08     0.30 1.00     2906     3218
## CRrate        0.06      0.09    -0.12     0.25 1.00     2158     2749
## meanRT       -0.00      0.00    -0.00     0.00 1.00     2674     3069
##
## Family Specific Parameters:
##       Estimate Est.Error l-95% CI u-95% CI Rhat Bulk_ESS Tail_ESS
## sigma     0.18      0.01     0.16     0.20 1.00     2962     2712
##
## Draws were sampled using sampling(NUTS). For each parameter, Bulk_ESS
## and Tail_ESS are effective sample size measures, and Rhat is the potential
## scale reduction factor on split chains (at convergence, Rhat = 1).
```

```
subj.resid.wm.outlier <- m.SV.wm.partial.outlier[["data"]][["subjectid"]] %>%
as_tibble()
colnames(subj.resid.wm.outlier) <- "subjectid"
res.WM.outlier <- resid(m.SV.wm.partial.outlier) %>% as_tibble() %>% select(E
stimate)
colnames(res.WM.outlier) <- "resid.wm"
res.subj.WM.outlier <- cbind(subj.resid.wm.outlier, res.WM.outlier)

#Speech Cog-ED
d.coged.speech.partial.outlier <- inner_join(d.coged.speech.partial, outlier.
subjs)

m.SV.speech.partial.outlier <- brm(data = d.coged.speech.partial.outlier, mea
nSV ~ taskCode +
                                     performance + (1 | subjectid),
                                  file = "models/m.SV.speech.partial.outlier
```

```
.rds")
summary(m.SV.speech.partial.outlier)

##  Family: gaussian
##   Links: mu = identity; sigma = identity
## Formula: meanSV ~ taskCode + performance + (1 | subjectid)
##    Data: d.coged.speech.partial.outlier (Number of observations: 297)
##   Draws: 4 chains, each with iter = 2000; warmup = 1000; thin = 1;
##          total post-warmup draws = 4000
##
## Group-Level Effects:
## ~subjectid (Number of levels: 99)
##               Estimate Est.Error l-95% CI u-95% CI Rhat Bulk_ESS Tail_ESS
## sd(Intercept)     0.20      0.02     0.16     0.25 1.00     1881     2884
##
## Population-Level Effects:
##             Estimate Est.Error l-95% CI u-95% CI Rhat Bulk_ESS Tail_ESS
## Intercept       0.35      0.13     0.08     0.61 1.00     2976     2978
## taskCode       -0.08      0.04    -0.15     0.00 1.00     3069     2826
## performance     0.00      0.00     0.00     0.01 1.00     2881     3361
##
## Family Specific Parameters:
##       Estimate Est.Error l-95% CI u-95% CI Rhat Bulk_ESS Tail_ESS
## sigma     0.24      0.01     0.21     0.26 1.00     3169     3319
##
## Draws were sampled using sampling(NUTS). For each parameter, Bulk_ESS
## and Tail_ESS are effective sample size measures, and Rhat is the potential
## scale reduction factor on split chains (at convergence, Rhat = 1).

subj.resid.speech.outlier <- m.SV.speech.partial.outlier[["data"]][["subjecti
d"]] %>% as_tibble()
colnames(subj.resid.speech.outlier) <- "subjectid"
res.speech.outlier <-residuals(m.SV.speech.partial.outlier) %>% as_tibble() %
>% select(Estimate)
colnames(res.speech.outlier) <- "resid.speech"
res.subj.Speech.outlier <- cbind(subj.resid.speech.outlier, res.speech.outlie
r)

SV.resids.outlier <- cbind(res.subj.WM.outlier, res.speech.outlier) %>% group
_by(subjectid) %>%
  summarise(mean.resid.wm = mean(resid.wm), mean.resid.speech = mean(resid.sp
eech))
#Testing for correlation between cognitive effort discounting across working 
memory & speech domains
#controlling for task performance
CogED.cor.partial.outlier <- cor_test(data = SV.resids.outlier, x = "mean.res
id.wm",
                                      y = "mean.resid.speech", bayesian = TRU
E,
```

```
                                      bayesian_prior = 0.707107)
```

When controlling for task difficulty and performance we still observed a positive correlation across working memory and speech comprehension domains, r = 0.37 [0.23, 0.50], BF_{10}= 230.53.

```
#Summarize Bayes Factor from correlation controlling for task performance
CogED.cor.partial.outlier
```

```
## Parameter1    |        Parameter2 |  rho |       95% CI |      pd | % in R
OPE |                Prior |         BF
## ---------------------------------------------------------------------------
-------------------------------------
## mean.resid.wm | mean.resid.speech | 0.37 | [0.23, 0.50] | 100%*** |     0.
20% | Beta (1.41 +- 1.41) | 230.53***
##
## Observations: 99
```

```
#Plot of correlation between working memory & speech comprehension domains co
ntrolling for task level & performance
fig.resid.outlier <- ggplot(SV.resids.outlier, aes(mean.resid.speech, mean.re
sid.wm)) +
   theme(plot.title = element_text(hjust = 0.5), panel.grid.major = element_b
lank(),
         panel.grid.minor = element_blank(), panel.background = element_blank
(),
         axis.line = element_line(colour = "black")) +
  geom_point() + geom_smooth(method=lm) +
  ggtitle("Controlling for task level & performance (reverse discounters remo
ved)") +
    xlab("Speech Comprehension") + ylab("Working Memory")
fig.resid.outlier
```

Here, we have plotted the residualized subjective value estimates, controlling for task load and performance after reverse discounters were removed.

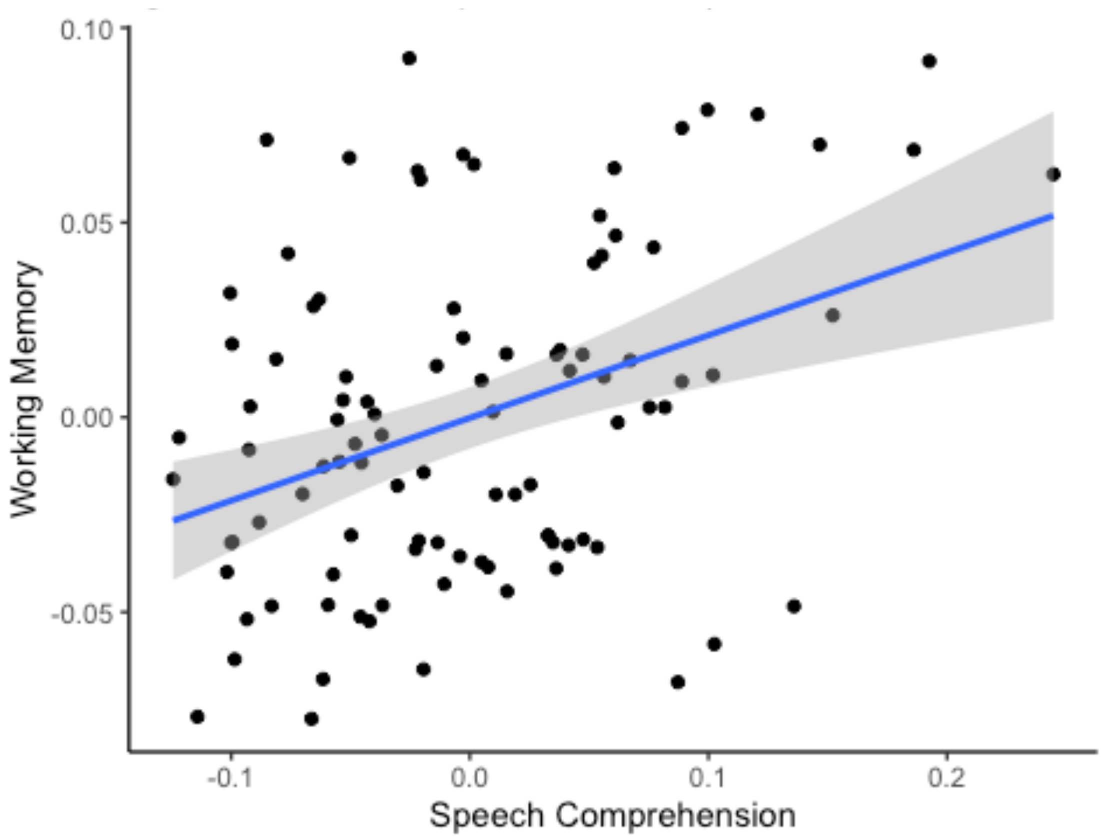

Partial correlation controlling for WMC and reward sensitivity (from residualized SV estimates)

For the final stage of analysis, we additionally controlled for individual differences in working memory capacity and reward sensitivity.

Reward Motivation (BIS/BAS, SPSRQ, GRAPES)

```
#BIS/BAS
#Importing and cleaning BIS/BAS
BISBAS.clean <- BISBAS %>% select(c(subjectid,completed, BAS_Drive, BAS_Fun,
BAS_Reward, BIS)) %>% distinct(subjectid, .keep_all=T) %>% mutate(BAS_total =
BAS_Drive + BAS_Fun + BAS_Reward)
BISBAS.SV <- inner_join(average.SV.outliers, BISBAS.clean)

#SPSRQ
#Importing and cleaning SPSRQ
SPSRQ.clean <- SPSRQ %>% select(c(subjectid, completed, SensitivityToReward,
SensitivityToPunishment)) %>% distinct(subjectid, .keep_all=T)
SPSRQ.SV <- inner_join(average.SV.outliers, SPSRQ.clean)

#GRAPES
GRAPES.clean <- GRAPES %>% select(c(subjectid, ends_with("response"))) %>% di
stinct(subjectid, .keep_all=T) %>%
  mutate(GRAPES.rew = q1_response + q4_response + q6_response + q7_response +
```

```
q9_response + q10_response + q15_response +
         q16_response + q16_response + q19_response + q20_response + q21_resp
onse + q25_response + q26_response + q27_response,
         GRAPES.pun = q2_response + q3_response + q5_response + q8_response +
q11_response + q12_response + q13_response +
         q14_response + q18_response + q22_response + q23_response + q24_resp
onse + q28_response + q29_response + q30_response)
GRAPES.SV <- inner_join(average.SV.outliers, GRAPES.clean)
```

Working Memory Capacity (Listing Span, Operation Span, Symmetry Span)

```
#Importing and cleaning WMC measures
#Listening Span
LSpan.clean <- LSpan %>% select(c(subjectid, completed, ListeningSpanScore))
%>%
  distinct(subjectid, .keep_all=T)
LSpan.SV <- inner_join(average.SV.outliers, LSpan.clean)

#Operation Span
OSpan.clean <- OSpan %>% select(c(subjectid, completed, ospan)) %>%
  distinct(subjectid, .keep_all=T)
OSpan.SV <- inner_join(average.SV.outliers, OSpan.clean)

#Symmetry Span
SymSpan.clean <- SymmSpan %>% select(c(subjectid, completed, sspan)) %>%
  distinct(subjectid, .keep_all=T)
SymSpan.SV <- inner_join(average.SV.outliers, SymSpan.clean)

#Create data frame with WMC measures, get z-scores, and create a composite me
asure
LSpan.comp <- LSpan.SV %>% select(-c(Speech, WM))
LSpan.comp$LSpan.z <- scale(LSpan.comp$ListeningSpanScore)
OSpan.comp <- OSpan.SV %>% select(-c(Speech, WM))
OSpan.comp$OSpan.z <- scale(OSpan.comp$ospan)
SymSpan.comp <- SymSpan.SV %>% select(-c(Speech, WM))
SymSpan.comp$SSpan.z <- scale(SymSpan.comp$sspan)

WMC.outlier <- inner_join(LSpan.comp, OSpan.comp, by = "subjectid") %>%
  inner_join(SymSpan.comp, by = "subjectid") %>%
  filter(completed.x == 1) %>% filter(completed.y == 1) %>% filter(completed
== 1) %>%
  select(-c("completed.x","completed.y","completed")) %>%
  mutate(WMC.composite = (LSpan.z + OSpan.z + SSpan.z)) %>%
  select(subjectid, WMC.composite)

#Create data frame with reward sensitivity measures, get z-scores, and create
a composite measure
BISBAS.comp <- BISBAS.SV %>% select(-c(Speech, WM, BAS_Drive, BAS_Fun, BAS_Re
ward, BIS))
BISBAS.comp$BAS.z <- scale(BISBAS.comp$BAS_total)
SPSRQ.comp <- SPSRQ.SV %>% select(-c(Speech, WM, SensitivityToPunishment))
```

```
SPSRQ.comp$SPSRQ.rew.z <- scale(SPSRQ.comp$SensitivityToReward)
GRAPES.comp <- GRAPES.SV %>% select(c(subjectid, GRAPES.rew))
GRAPES.comp$GRAPES.rew.z <- scale(GRAPES.comp$GRAPES.rew)

Reward.composite.outlier <- inner_join(BISBAS.comp, SPSRQ.comp, by = "subject
id") %>%
  inner_join(GRAPES.comp, by = "subjectid") %>%
  filter(completed.x == 1) %>% filter(completed.y == 1) %>%
  select(-c("completed.x","completed.y")) %>%
  mutate(Rew.composite = (BAS.z + SPSRQ.rew.z + GRAPES.rew.z)) %>%
  select(subjectid, Rew.composite)
```

Plotting Distributions of Composite Measures

```
#WMC density plot
WMC.density <- ggplot(WMC.outlier, aes(x=WMC.composite))+
  geom_density(color="darkblue", fill="lightblue")

#Reward density plot
Reward.density <- ggplot(Reward.composite.outlier, aes(x=Rew.composite))+
  geom_density(color="darkred", fill="pink")
```

Partial correlation controlling for WMC and reward sensitivity (from residualized SV estimates)

After additionally controlling for individual differences in working memory capacity and reward sensitivity we observed a positive correlation across working memory and speech comprehension domains, r = 0.39 [0.26, 0.53], BF_{10}= 563.18.

```
#Testing for partial correlation between residuals (from stage two) controlli
ng for WMC and reward sensitivity
SV.composite.resid.outlier <- inner_join(WMC.outlier, Reward.composite.outlie
r, by = "subjectid") %>%
  inner_join(SV.resids.outlier, by = "subjectid")
SV.composite.resid.outlier.clean <- cbind(SV.composite.resid.outlier$WMC.comp
osite,
                                          SV.composite.resid.outlier$Rew.comp
osite,
                                          SV.composite.resid.outlier$mean.res
id.speech,
                                          SV.composite.resid.outlier$mean.res
id.wm) %>% as_tibble()
colnames(SV.composite.resid.outlier.clean) <- c("WMC", "Reward", "Speech", "W
M")

WMC.resid.cor.outlier <- cor_test(data = SV.composite.resid.outlier.clean, x
= "WM", y = "Speech",
                                  bayesian = TRUE, partial_bayesian = TRUE, b
ayesian_prior  = 0.707107)
#Summarize Bayes Factor from correlation
WMC.resid.cor.outlier
```

```
## Parameter1 | Parameter2 |  rho |       95% CI |      pd | % in ROPE |
Prior |        BF
## ----------------------------------------------------------------------
---------------------------
## WM         |     Speech | 0.39 | [0.24, 0.52] | 100%*** |     0.07% | Beta
(1.41 +- 1.41) | 563.14***
##
## Observations: 99

#WM Cog-ED
d.coged.wm.partial.outlier.plot <- inner_join(d.coged.wm.partial.outlier, WMC
.outlier,
                                              by = "subjectid") %>%
  inner_join(Reward.composite.outlier, by = "subjectid")
m.SV.wm.partial.outlier.plot <- brm(data = d.coged.wm.partial.outlier.plot, m
eanSV ~ taskCode +
                                    hitrate + CRrate + meanRT + WMC.composite
+ Rew.composite + (1 | subjectid),
                                    file = "models/m.SV.wm.partial.outlier.pl
ot.rds")

subj.resid.wm.outlier.plot <- m.SV.wm.partial.outlier.plot[["data"]][["subjec
tid"]] %>% as_tibble()
colnames(subj.resid.wm.outlier.plot) <- "subjectid"
res.WM.outlier.plot <- residuals(m.SV.wm.partial.outlier.plot) %>% as_tibble(
) %>% select(Estimate)
colnames(res.WM.outlier.plot) <- "resid.wm"
res.subj.WM.outlier.plot <- cbind(subj.resid.wm.outlier.plot, res.WM.outlier.
plot)

#Speech Cog-ED
d.coged.speech.partial.outlier.plot <- inner_join(d.coged.speech.partial.outl
ier, WMC.outlier,
                                                  by = "subjectid") %>%
  inner_join(Reward.composite.outlier, by = "subjectid")
m.SV.speech.partial.outlier.plot <- brm(data = d.coged.speech.partial.outlier
.plot, meanSV ~ taskCode +
                                          performance + WMC.composite + Rew.c
omposite + (1 | subjectid),
                                        file = "models/m.SV.speech.partial.ou
tlier.plot.rds")

subj.resid.speech.outlier.plot <- m.SV.speech.partial.outlier.plot[["data"]][
["subjectid"]] %>% as_tibble()
colnames(subj.resid.speech.outlier.plot) <- "subjectid"
res.speech.outlier.plot <-residuals(m.SV.speech.partial.outlier.plot) %>% as_
tibble() %>%
  select(Estimate)
colnames(res.speech.outlier.plot) <- "resid.speech"
res.subj.Speech.outlier.plot <- cbind(subj.resid.speech.outlier.plot, res.spe
```

```
ech.outlier.plot)

SV.resids.outlier.plot <- cbind(res.subj.WM.outlier.plot, res.speech.outlier.
plot) %>%
  group_by(subjectid) %>%
  summarise(mean.resid.wm = mean(resid.wm), mean.resid.speech = mean(resid.sp
eech))
#Testing for correlation between cognitive effort discounting across working
memory & speech domains
#controlling for task performance
CogED.cor.partial.outlier.plot <- cor_test(data = SV.resids.outlier.plot,
                                           x = "mean.resid.wm", y = "mean.res
id.speech",
                                           bayesian = TRUE, bayesian_prior =
0.707107)

#Plot of correlation between working memory & speech comprehension domains
#controlling for task level, performance, WMC, and reward sensitivity
fig.resid.outlier.plot <- ggplot(SV.resids.outlier.plot, aes(mean.resid.speec
h, mean.resid.wm)) +
   theme(plot.title = element_text(hjust = 0.5), panel.grid.major = element_b
lank(),
         panel.grid.minor = element_blank(), panel.background = element_blank
(),
         axis.line = element_line(colour = "black")) +
  geom_point() + geom_smooth(method=lm) +
  ggtitle("") +
    xlab("Residualized Subjective Value: Speech Comprehension") + ylab("Resid
ualized Subjective Value: Working Memory")
fig.resid.outlier.plot
```

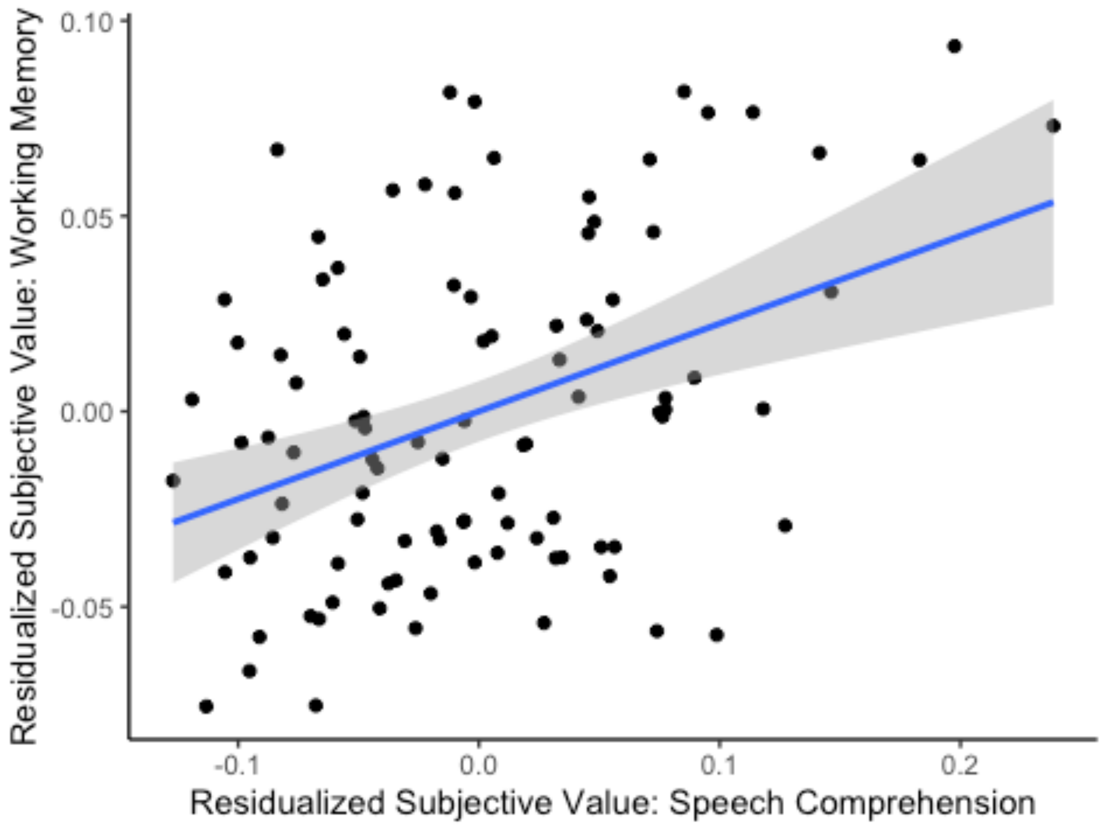


```
ggsave(fig.resid.outlier.plot, filename = "Figure2c.pdf", width = 10)
```

NASA TLX

Participants completed NASA ratings after each load level during the familiarization phase (likert scale: 1-21; higher values indicate greater endorsement)

As reported in the main text, there was a main effect of task load across ratings of mental demand ß= 3.64 [2.94, 4.31], SD=0.35, effort ß = 2.52 [1.93, 3.14], SD=0.31, and frustration ß = 0.98 [0.20, 1.73], SD=0.39. In addition, there was also a main effect of domain for self-reported ratings of mental demand, ß = 3.10 [1.88, 4.30], SD=0.61, and effort, ß = 2.65 [1.62, 3.73], SD=0.54., such that ratings of subjective mental demand and effort were greater for the speech comprehension task, relative to the working memory task. Frustration ratings did not differ across task domain, ß = 0.35 [-0.95, 1.67], SD=0.68. Finally, there was an interaction between task load and domain across ratings of mental demand, ß = -0.57 [-0.99, -0.13], SD=0.22, and frustration, ß = 0.72 [0.24, 1.20], SD=0.25. There was no interaction between task load and domain for ratings of effort, ß = -0.34 [-0.74, 0.04], SD=0.20.

```
#Mental Demand Ratings
NASA.m.demand.wm <- coged.wm %>% select(subjectid, completed, mentaldemand_1,
                                        mentaldemand_2, mentaldemand_3, menta
ldemand_4) %>%
```

```
  group_by(subjectid) %>%
  filter(completed == 1) %>%
  mutate(Domain = "WM")

NASA.m.demand.speech <- coged.speech %>% select(subjectid, completed, mentald
emand_1,
                                                mentaldemand_2, mentaldemand_
3, mentaldemand_4) %>%
  group_by(subjectid) %>%
  filter(completed == 1) %>%
  mutate(Domain = "Speech")

NASA.m.demand <- rbind(NASA.m.demand.wm,NASA.m.demand.speech) %>% select(-com
pleted) %>%
  pivot_longer(names_to = "mental_demand", values_to = "rating", -c(subjectid
,Domain)) %>%
  separate(col = mental_demand, into=c(NA,"Task"), sep = "_") %>%
  mutate(taskCode = factor(Task, levels=c(1,2,3,4), labels=c(0,1,2,3)),
         domainCode = factor(Domain, levels = c("WM","Speech"), labels = c(0
,1)))

NASA.m.demand$taskCode <- as.numeric(NASA.m.demand$taskCode)
NASA.m.demand$domainCode <- as.numeric(NASA.m.demand$domainCode)
NASA_mdemand_sum <- summarySEwithin2(NASA.m.demand, measurevar = "rating",
                                     withinvars = c("Task","Domain"), idvar =
"subjectid")
NASA_mdemand_sum$Task <- factor(NASA_mdemand_sum$Task, levels = c(1,2,3,4),
                                labels = c("black","red","blue","purple"))

m.mentalDemand <- brm(data = NASA.m.demand, rating ~ taskCode*domainCode + (1
| subjectid),
                      file = "models/m.mentalDemand.rds")
summary(m.mentalDemand)

##  Family: gaussian
##   Links: mu = identity; sigma = identity
## Formula: rating ~ taskCode * domainCode + (1 | subjectid)
##    Data: NASA.m.demand (Number of observations: 856)
##   Draws: 4 chains, each with iter = 2000; warmup = 1000; thin = 1;
##          total post-warmup draws = 4000
##
## Group-Level Effects:
## ~subjectid (Number of levels: 107)
##               Estimate Est.Error l-95% CI u-95% CI Rhat Bulk_ESS Tail_ESS
## sd(Intercept)     2.65      0.23     2.23     3.12 1.00     1148     1789
##
## Population-Level Effects:
##                     Estimate Est.Error l-95% CI u-95% CI Rhat Bulk_ESS Tai
l_ESS
## Intercept               3.50      0.97     1.58     5.41 1.00     2127
```

```
2549
## taskCode                 3.69      0.35     3.04     4.39 1.00     2195
2274
## domainCode               3.09      0.60     1.91     4.27 1.00     2178
2553
## taskCode:domainCode     -0.59      0.22    -1.03    -0.17 1.00     2134
2405
##
## Family Specific Parameters:
##       Estimate Est.Error l-95% CI u-95% CI Rhat Bulk_ESS Tail_ESS
## sigma     3.65      0.09     3.47     3.84 1.00     4122     2960
##
## Draws were sampled using sampling(NUTS). For each parameter, Bulk_ESS
## and Tail_ESS are effective sample size measures, and Rhat is the potential
## scale reduction factor on split chains (at convergence, Rhat = 1).
```

```
p.m.demand <- ggplot(NASA_mdemand_sum, aes(x=Task, y=rating, fill=Domain)) +
  theme(plot.title = element_text(hjust = 0.5), panel.grid.major = element_bl
ank(),
        panel.grid.minor = element_blank(), panel.background = element_blank(
),
        axis.line = element_line(colour = "black")) +
  geom_bar(stat="identity", position=position_dodge()) +
  geom_errorbar(position=position_dodge(width=0.9), aes(ymin=rating-ci, ymax=
rating+ci), width=.2) +
  xlab("Task") + ylab("Mental Demand") + ggtitle("Self-Reported Mental Demand
")
p.m.demand + labs(fill = "Domain") + scale_fill_brewer(palette = "Paired")
```

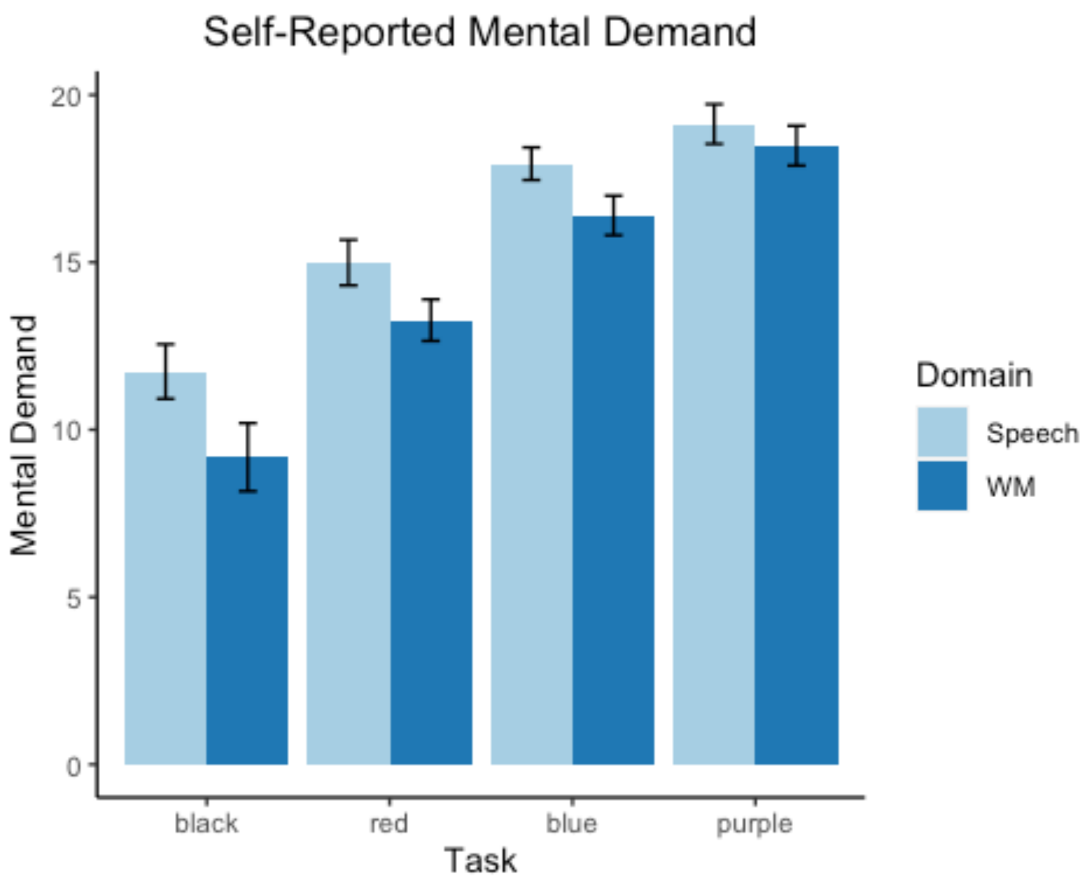


```
#Frustration Ratings
NASA.frust.wm <- coged.wm %>% select(subjectid, completed, frustration_1,
                                     frustration_2, frustration_3, frustratio
n_4) %>%
  group_by(subjectid) %>%
  filter(completed == 1) %>%
  mutate(Domain = "WM")

NASA.frust.speech <- coged.speech %>% select(subjectid, completed, frustratio
n_1,
                                             frustration_2, frustration_3, fr
ustration_4) %>%
  group_by(subjectid) %>%
  filter(completed == 1) %>%
  mutate(Domain = "Speech")

NASA.frust <- rbind(NASA.frust.wm,NASA.frust.speech) %>% select(-completed) %
>%
  pivot_longer(names_to = "frustration", values_to = "rating", -c(subjectid,
Domain)) %>%
  separate(col = frustration, into=c(NA,"Task"), sep = "_") %>%
  mutate(taskCode = factor(Task, levels=c(1,2,3,4), labels=c(0,1,2,3)),
         domainCode = factor(Domain, levels = c("WM","Speech"), labels = c(0
```

```
,1)))
NASA.frust$taskCode <- as.numeric(NASA.frust$taskCode)
NASA.frust$domainCode <- as.numeric(NASA.frust$domainCode)

m.Frustration <- brm(data = NASA.frust, rating ~ taskCode*domainCode + (1 | s
ubjectid),
                     file = "models/m.Frustration.rds")
summary(m.Frustration)

##  Family: gaussian
##   Links: mu = identity; sigma = identity
## Formula: rating ~ taskCode * domainCode + (1 | subjectid)
##    Data: NASA.frust (Number of observations: 856)
##   Draws: 4 chains, each with iter = 2000; warmup = 1000; thin = 1;
##          total post-warmup draws = 4000
##
## Group-Level Effects:
## ~subjectid (Number of levels: 107)
##               Estimate Est.Error l-95% CI u-95% CI Rhat Bulk_ESS Tail_ESS
## sd(Intercept)     4.01      0.32     3.45     4.68 1.01      756     1000
##
## Population-Level Effects:
##                     Estimate Est.Error l-95% CI u-95% CI Rhat Bulk_ESS Tai
l_ESS
## Intercept               6.81      1.19     4.55     9.16 1.00     1459
1982
## taskCode                1.04      0.41     0.24     1.83 1.00     1869
2464
## domainCode              0.43      0.70    -0.94     1.77 1.00     1977
2326
## taskCode:domainCode     0.68      0.26     0.18     1.18 1.00     1909
2505
##
## Family Specific Parameters:
##       Estimate Est.Error l-95% CI u-95% CI Rhat Bulk_ESS Tail_ESS
## sigma     4.11      0.11     3.91     4.32 1.00     3178     2981
##
## Draws were sampled using sampling(NUTS). For each parameter, Bulk_ESS
## and Tail_ESS are effective sample size measures, and Rhat is the potential
## scale reduction factor on split chains (at convergence, Rhat = 1).

NASA_frust_sum <- summarySEwithin2(NASA.frust, measurevar = "rating",
                                   withinvars = c("Task","Domain"), idvar = "
subjectid")
NASA_frust_sum$Task <- factor(NASA_frust_sum$Task, levels = c(1,2,3,4),
                              labels = c("black","red","blue","purple"))

p.frust <- ggplot(NASA_frust_sum, aes(x=Task, y=rating, fill=Domain)) +
  theme(plot.title = element_text(hjust = 0.5), panel.grid.major = element_bl
ank(),
```

```
        panel.grid.minor = element_blank(), panel.background = element_blank(
),
        axis.line = element_line(colour = "black")) +
  geom_bar(stat="identity", position=position_dodge()) +
  geom_errorbar(position=position_dodge(width=0.9), aes(ymin=rating-ci, ymax=
rating+ci), width=.2) +
  xlab("Task") + ylab("Frustration") + ggtitle("Self-Reported Frustration")
p.frust +  labs(fill = "Domain") + scale_fill_brewer(palette = "Paired")
```

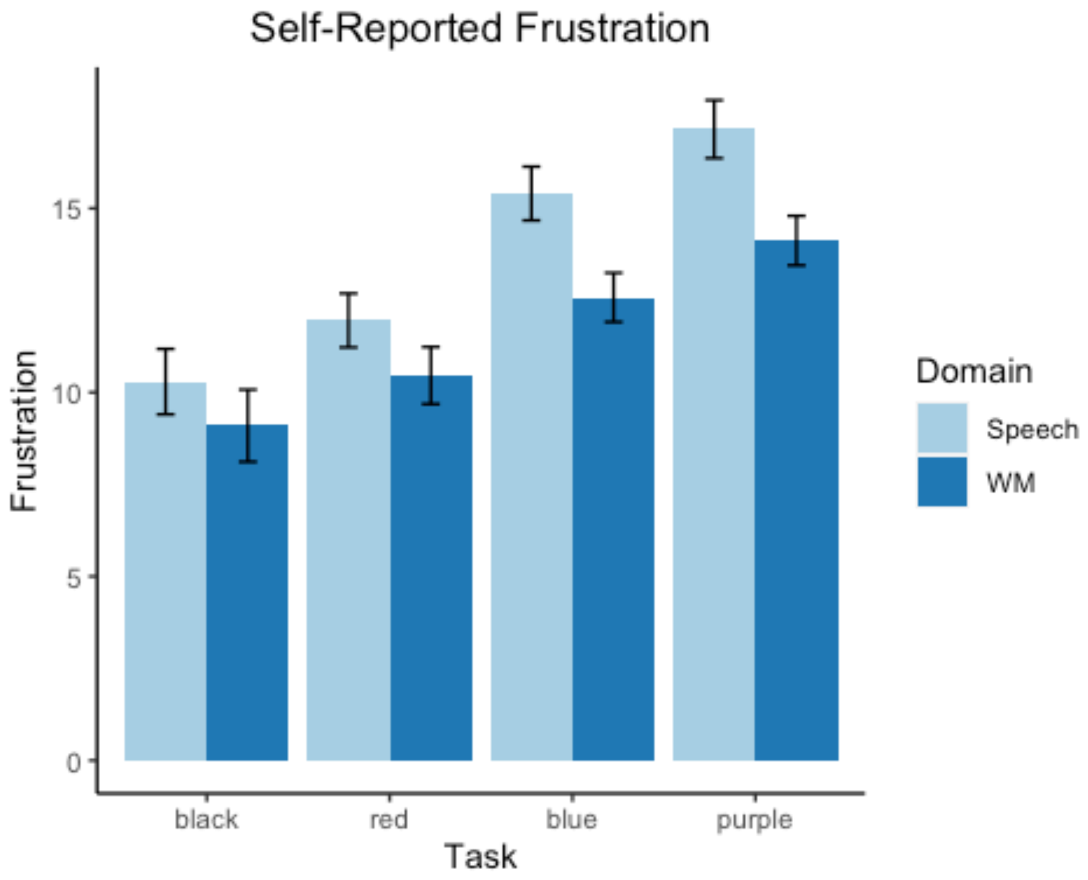


```
#Effort Ratings
NASA.effort.wm <- coged.wm %>% select(subjectid, completed, effort_1,
                                      effort_2, effort_3, effort_4) %>%
  group_by(subjectid) %>%
  filter(completed == 1) %>%
  mutate(Domain = "WM")

NASA.effort.speech <- coged.speech %>% select(subjectid, completed, effort_1,
                                              effort_2, effort_3, effort_4) %
>%
  group_by(subjectid) %>%
  filter(completed == 1) %>%
  mutate(Domain = "Speech")
```

```
NASA.effort <- rbind(NASA.effort.wm,NASA.effort.speech) %>% select(-completed
) %>%
  pivot_longer(names_to = "effort", values_to = "rating", -c(subjectid,Domain
)) %>%
  separate(col = effort, into=c(NA,"Task"), sep = "_") %>%
    mutate(taskCode = factor(Task, levels=c(1,2,3,4), labels=c(0,1,2,3)),
          domainCode = factor(Domain, levels = c("WM","Speech"), labels = c(0
,1)))
NASA.effort$taskCode <- as.numeric(NASA.effort$taskCode)
NASA.effort$domainCode <- as.numeric(NASA.effort$domainCode)

m.Effort <- brm(data = NASA.effort, rating ~ taskCode*domainCode + (1 | subje
ctid),
                file = "models/m.Effort.rds")
summary(m.Effort)

##  Family: gaussian
##   Links: mu = identity; sigma = identity
## Formula: rating ~ taskCode * domainCode + (1 | subjectid)
##    Data: NASA.effort (Number of observations: 856)
##   Draws: 4 chains, each with iter = 2000; warmup = 1000; thin = 1;
##          total post-warmup draws = 4000
##
## Group-Level Effects:
## ~subjectid (Number of levels: 107)
##                 Estimate Est.Error l-95% CI u-95% CI Rhat Bulk_ESS Tail_ESS
## sd(Intercept)       2.79      0.23     2.37     3.27 1.00     1025     1820
##
## Population-Level Effects:
##                       Estimate Est.Error l-95% CI u-95% CI Rhat Bulk_ESS Tai
l_ESS
## Intercept                 6.39      0.92     4.57     8.19 1.00     1940
2231
## taskCode                  2.52      0.32     1.89     3.16 1.00     2037
2493
## domainCode                2.55      0.56     1.46     3.67 1.00     2051
2209
## taskCode:domainCode      -0.33      0.21    -0.74     0.07 1.00     2004
2224
##
## Family Specific Parameters:
##       Estimate Est.Error l-95% CI u-95% CI Rhat Bulk_ESS Tail_ESS
## sigma     3.32      0.09     3.16     3.50 1.00     4039     3217
##
## Draws were sampled using sampling(NUTS). For each parameter, Bulk_ESS
## and Tail_ESS are effective sample size measures, and Rhat is the potential
## scale reduction factor on split chains (at convergence, Rhat = 1).

NASA_effort_sum <- summarySEwithin2(NASA.effort, measurevar = "rating",
                                    withinvars = c("Task","Domain"), idvar =
```

```
"subjectid")
NASA_effort_sum$Task <- factor(NASA_effort_sum$Task, levels = c(1,2,3,4),
                               labels = c("black","red","blue","purple"))

p.effort <- ggplot(NASA_effort_sum, aes(x=Task, y=rating, fill=Domain)) +
  theme(plot.title = element_text(hjust = 0.5), panel.grid.major = element_bl
ank(),
        panel.grid.minor = element_blank(), panel.background = element_blank(
),
        axis.line = element_line(colour = "black")) +
  geom_bar(stat="identity", position=position_dodge()) +
  geom_errorbar(position=position_dodge(width=0.9), aes(ymin=rating-ci, ymax=
rating+ci), width=.2) +
  xlab("Task") + ylab("Effort") + ggtitle("Self-Reported Effort")
p.effort + labs(fill = "Domain") + scale_fill_brewer(palette = "Paired")
```

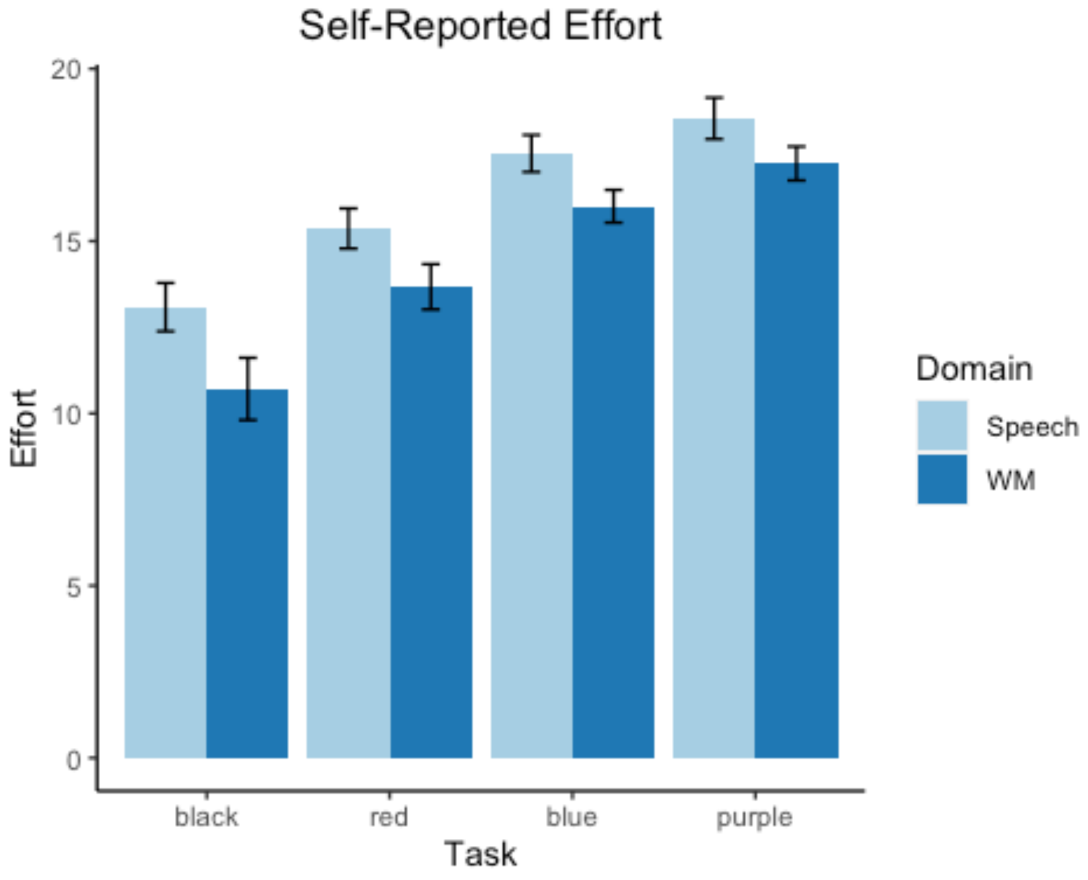

Supplement: Supplementary file 2 — Additional file 2. Supplementary material for Main Text. [file 41235_2022_363_MOESM2_ESM.pdf]
